# Disclosing the Role of C4-Oxo Substitution in the Photochemistry of DNA and RNA Pyrimidine Monomers: Formation of Photoproducts from the Vibrationally Excited Ground State

**DOI:** 10.1021/acs.jpclett.2c00052

**Published:** 2022-02-22

**Authors:** Eva Vos, Sean J. Hoehn, Sarah E. Krul, Carlos E. Crespo-Hernández, Jesús González-Vázquez, Inés Corral

**Affiliations:** † Departamento de Química, Módulo 13, Universidad Autónoma de Madrid, 28049 Madrid, Spain; ‡ Department of Chemistry, Case Western Reserve University, 10900 Euclid Avenue, Cleveland, Ohio 44106, United States; ∇ Institute for Advanced Research in Chemistry (IAdChem), Universidad Autónoma de Madrid, 28049 Madrid, Spain

## Abstract

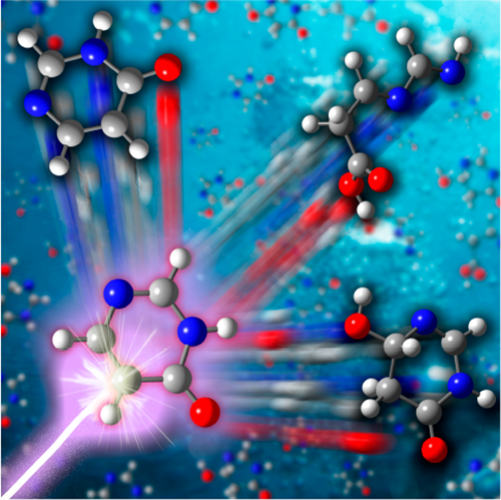

Oxo and amino substituted purines and pyrimidines have been suggested as protonucleobases participating in ancient pre-RNA forms. Considering electromagnetic radiation as a key environmental selection pressure on early Earth, the investigation of the photophysics of modified nucleobases is crucial to determine their viability as nucleobases’ ancestors and to understand the factors that rule the photostability of natural nucleobases. In this Letter, we combine femtosecond transient absorption spectroscopy and quantum mechanical simulations to reveal the photochemistry of 4-pyrimidinone, a close relative of uracil. Irradiation of 4-pyrimidinone with ultraviolet radiation populates the S_1_(ππ*) state, which decays to the vibrationally excited ground state in a few hundred femtoseconds. Analysis of the postirradiated sample in water reveals the formation of a 6-hydroxy-5H-photohydrate and 3-(*N*-(iminomethyl)imino)propanoic acid as the primary photoproducts. 3-(*N*-(Iminomethyl)imino)propanoic acid originates from the hydrolysis of an unstable ketene species generated from the C4–N3 photofragmentation of the pyrimidine core.

Modified nucleobases are important (i) in natural RNA, where they assist in the control of the stability of the macrostructure and in the regulation of translation and recognition processes;^1^ (ii) in artificial genetic biopolymers, sought for understanding the working mechanism, modifying the natural functionality, and multiplying the possibilities for information storage of natural DNA and RNA;^2−9^ and (iii) in the field of prebiotic chemistry, where they have been proposed as predecessors of the extant natural nucleobases.^10,11^

The noncanonical nucleobase 4-pyrimidinone (4OPy), whose photophysics and photochemistry is investigated in this Letter, is a close relative of uracil reduced at the C2-position. This modified nucleobase has been proposed together with 2-thio-iso-guanine as a nonstandard Watson–Crick base pair, successfully recognized and copied by polymerase.^12^ Interestingly, 4OPy also appears in the list of potential nucleobase ancestors suggested by Cafferty and Hud^10^ and has been identified in model prebiotic reactions.^13−16^ In fact, 4OPy was identified, in significant amounts, as a product of the condensation reaction of formamide catalyzed by cosmic dust analogues^15^ or alumina and silica, both used as model inorganic oxides present on early Earth.^16^ Importantly, 4OPy was also detected by liquid and gas chromatography in the light-mediated reaction of H_2_O:pyrimidine,^14^ NH_3_:pyrimidine, and H_2_O:NH_3_:pyrimidine ice mixtures.^13^ Calculations in the frame of density functional and second-order perturbation theory suggest the formation of 4OPy from the reaction of OH radicals with ionized pyrimidine radical cations and the subsequent release of a proton to the solvent bulk from the intermediate 4-hydroxypyrimidine.^17^ Uracil formation, also observed in the experiments in refs (13 and 14), in turn, is predicted to occur from the subsequent attack of OH radicals to 4-hydroxypyrimidine and 4OPy.

In this Letter, steady-state and time-resolved spectroscopy, the static mapping of the potential energy surfaces (PES), and molecular dynamics (MD) simulations are combined to investigate the photochemistry of 4OPy to (i) evaluate its viability as a nucleobase ancestor and (ii) establish the influence of the substituents on the photochemistry of pyrimidines.

As a first step, we have assigned the low-energy region of the absorption spectrum of 4-(3H)-pyrimidinone, the only tautomer predicted to be available at room temperature (see the Supporting Information). The experimental absorption spectra of 4OPy recorded in acetonitrile (ACN) and in aqueous solution pH 7.4 (PBS), see Figure 1, consist of two absorption bands at 260 and 214 nm in ACN (250 and 222 nm in PBS). The least energetic absorption band is assigned to a combination of the S_1_ and S_2_ states, with a predominant ππ* character and contributions from n_N_π* excitations. The second absorption band, also with a predominant ππ* character, is ascribed to the superposition of the S_4_ and S_5_ states.

**Figure 1:**
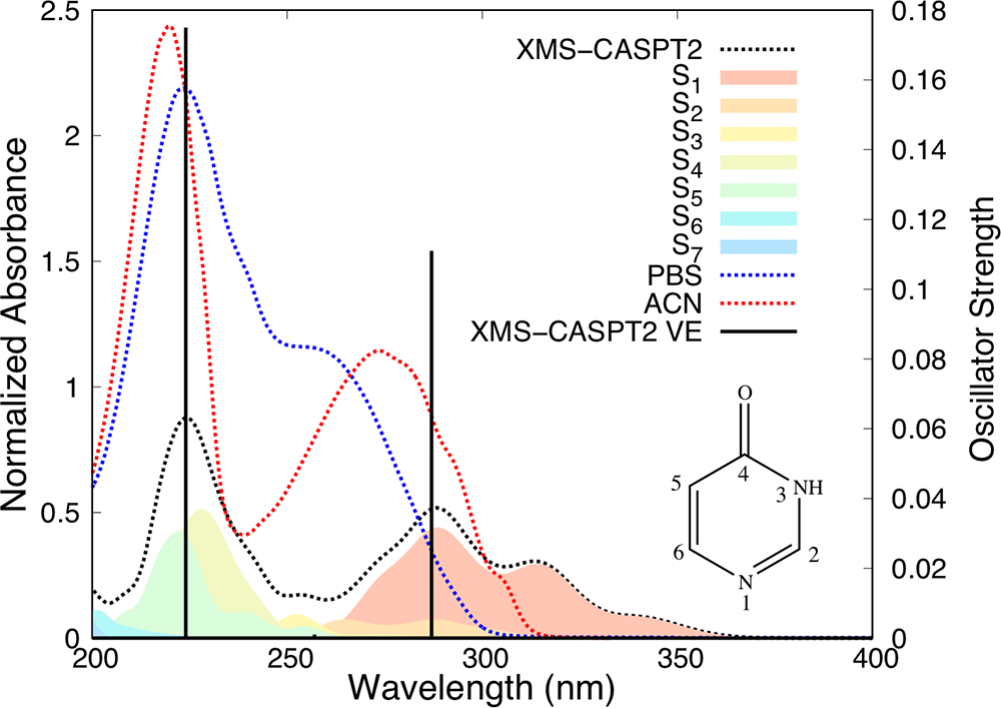
Experimental (PBS (blue) and ACN (red)) and gas-phase semiclassical (black dotted line; S_1_–S_7_ excitation contributions in different colors) absorption spectra of 4OPy. Black vertical lines represent the XMS-CASPT2 vertical excitations (Table S4).

Femtosecond broadband transient absorption spectroscopy (TAS) was used to probe the excited-state dynamics of 4OPy upon 267 nm excitation in ACN and PBS. In both solvents, a transient species is observed within the cross correlation of the pump and probe beams with a maximum at 320 nm and a broad tail of lesser intensity extending out to 700 nm (Figures 2a and S1a). Within the following ca. 400 fs (Figure 2b), a decrease in absorbance from ca. 450 to 700 nm is observed in ACN, while a simultaneous increase in absorbance occurs from 320 to 450 nm. The UV transient species decays uniformly within ca. 30 ps in ACN (Figures 2c) and in less than 5 ps in PBS (Figures S1b). Similar transient absorption dynamics was observed following excitation at 290 nm, as shown in Figure S5.

**Figure 2:**
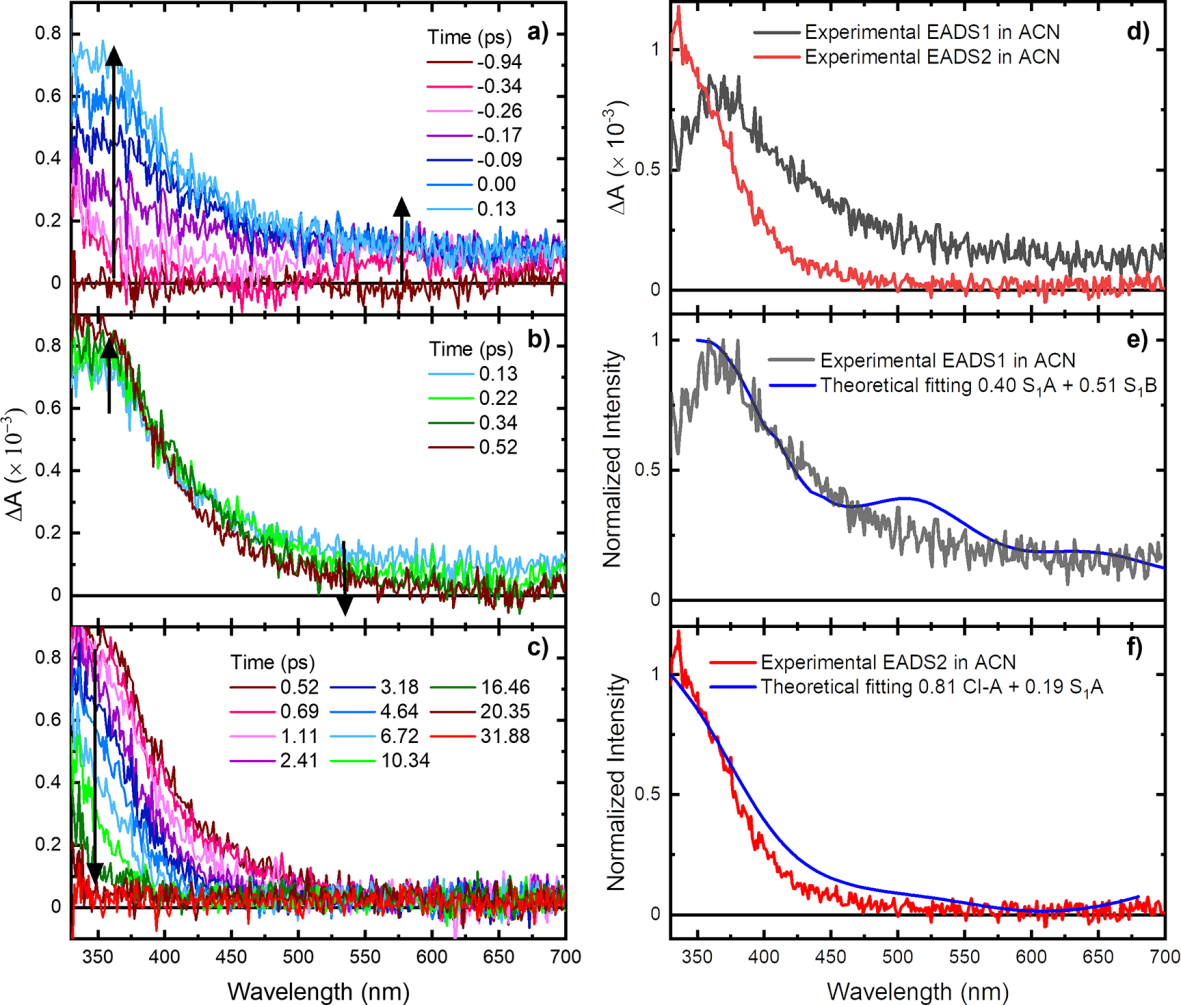
TAS of 4OPy in ACN (a–c) following excitation at 267 nm. Evolution associated difference spectra (EADS) obtained from global and target analyses with a two-component sequential model in ACN (d). Superposition of experimental (black and red lines) and simulated EADS (blue line) in ACN (e and f). The simulated e and f spectra were shifted by +0.68 eV and −0.2 eV.

The full broadband data can be fit with a two-component sequential model for both solvents at both excitation wavelengths. In ACN, the global “average” lifetimes were found to be τ_1_ = 0.8 ± 0.1 ps and τ_2_ = 8.5 ± 0.3 ps. For PBS, ultrafast lifetimes τ_1_ < 0.25 ps (267 nm) and τ_1_ = 0.5 ± 0.1 ps (290 nm) and a global “average” τ_2_ lifetime of 1.1 ± 0.2 ps at both excitation wavelengths were obtained. Evolution associated difference spectra (EADS) and representative kinetic traces extracted from the global and target analyses following excitation at 267 nm in ACN and PBS are shown in Figures 2d and S2 (Figure S6 for 290 nm excitation).

Further insight into the relaxation mechanism can be gained from exploring the topography of the excited and S_0_ PES of 4OPy, the simulation of the TAS, and the output of MD simulations. Figure 3a sketches the main topological features of the 4OPy PES relevant to its main deactivation route. Minimum energy path calculations starting from the Franck–Condon (FC) region of the S_1_ state (the main contributor to the first band in the absorption spectrum and predominant electronic state populated after excitation at 267 nm) locate a ππ* minimum, S_1_*A*_min_, at 3.69 eV above the S_0_ minimum. This minimum loses the characteristic planarity of the FC geometry and presents a C2 puckered structure (Figure S12). We find a second isoenergetic minimum, S_1_*B*_min_, also puckered at the C2 position, which additionally uplifts the H atom sitting at this center with respect to its original position. A transition state (TS) of only a few millielectronvolts (0.02 eV) separates these two minima. Decay from these two minima to the S_0_ is possible via two energetically accessible degeneracy regions located at 3.85 and 3.70 eV, CI–A_S1/S0/T1_ and CI–B_S1/S0/T1_, geometrically very similar to the S_1_*A*_min_ and S_1_*B*_min_ minima, respectively. A similar S_1_/S_0_ crossing for this system was found by Delchev et al.^18^ The minima are separated from the internal conversion (IC) funnels by slightly upward potential energy profiles (Figure 3a).

**Figure 3:**
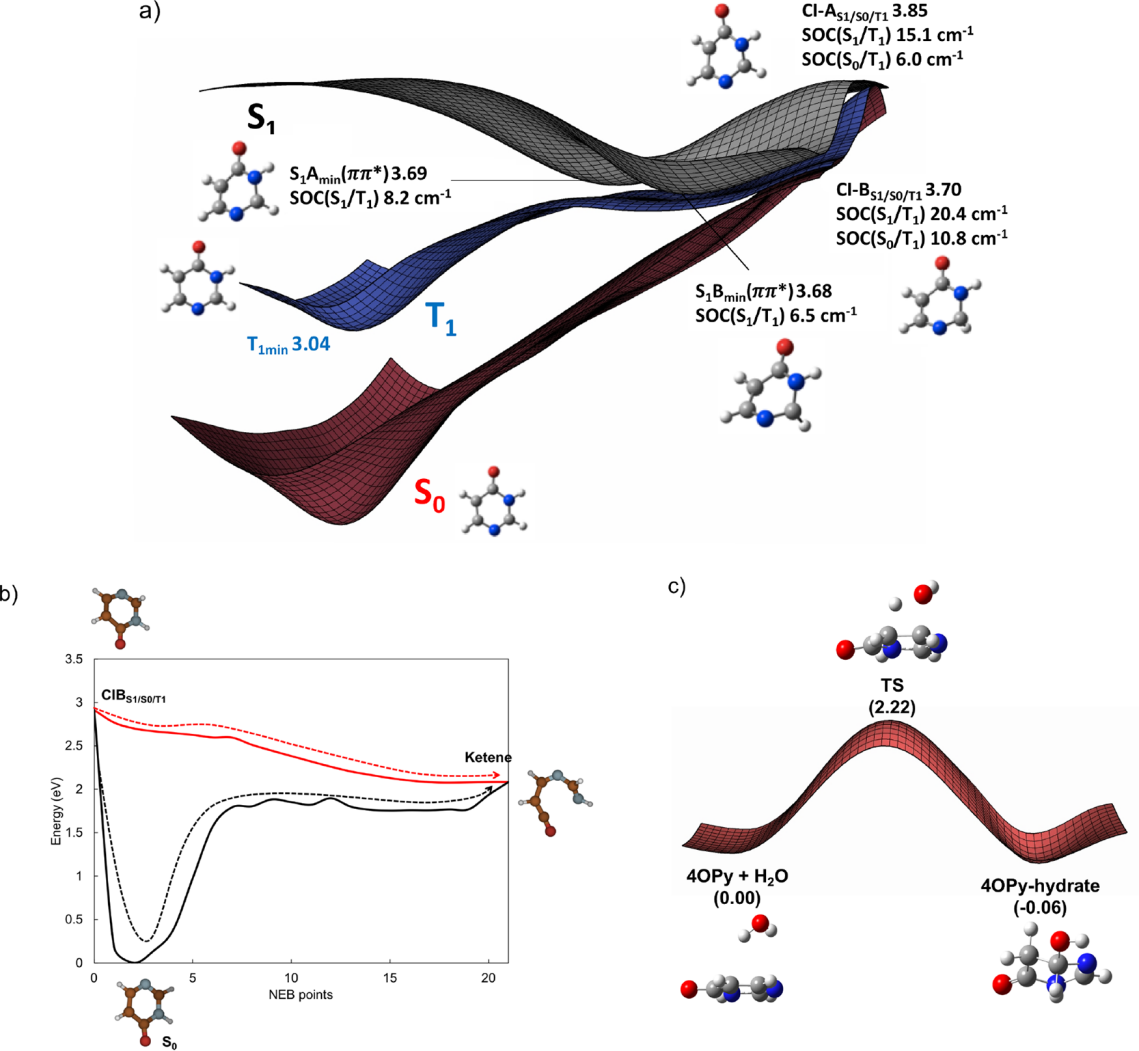
(a) Key features of the XMS-CASPT2 PES of 4OPy along the coordinate relevant to its decay. Energies in eV relative to the S_0_ minimum. (b) XMS-CASPT2 interpolated (red) and optimized minimum energy (black) S_0_ paths connecting the CI–B_S1/S0/T1_ with the ketene minimum obtained via nudged elastic band (NEB) calculation. (c) XMS-CASPT2 S_0_ PES for 4OPy Hydration.

Given these results, we propose the following as the preferred competing deactivation routes: S_1_* → S_1_*A*_min_ → (i) CI-A_S1/S0/T1_ → S_0_; (ii) TS → S_1_*B*_min_ → CI–B_S1/S0/T1_ → S_0_. Alternative minor deactivation routes along the triplet manifold were also investigated and are reported in the Supporting Information. Support for this mechanism is provided by the interpretation of the experimental TAS (Figures 2a–c and S1) and EADS (Figures 2d and S2). For this, we have computed the absorption spectra at key regions of the excited PES of Figures 3a and S11, where the system is expected to access along the deactivation mechanism. These spectra were linearly combined to provide a semiquantitative interpretation to the extracted EADS in ACN and PBS. At 267 nm, we find that the two species contributing predominantly to EADS1 in ACN (Figure 2d) and PBS (Figure S2b) are the S_1_*A*_min_ and the S_1_*B*_min_ minima (Figures 2e and S3a), fully consistent with the mechanism predicted by the static mapping of the PES. Therefore, population of the S_1_ minima is proposed to occur within our instrument response (IRF) of 250 ± 50 fs. Then, the population movement from the S_1_ minima to the vibrationally hot S_0_, via S_1_/S_0_ CIs, is assigned to τ_1_ = 0.8 ± 0.1 ps (ACN) and to τ_1_ < 0.25 ps in PBS. This is supported by the good agreement between the EADS2 (Figures 2d and S2b) and the simulated signal which combines the absorption from CI_S1/S0/T1_ and residual absorption from the S_1 min_ (Figures 2f and S3b). Lastly, the hot S_0_ is proposed to vibrationally relax with an average lifetime of 8.4 ± 0.2 ps in ACN and 1.2 ± 0.2 ps in PBS. As shown in Table S1, with a lower excitation energy (290 nm), the excited-state population gets trapped in the S_1_ minima for longer, resulting in a lifetime of τ_1_ 1.0 ± 0.1 ps (ACN) and 0.5 ± 0.1 ps (PBS) for S_1_ to S_0_ IC. The hot S_0_ is proposed to vibrationally relax with a lifetime of 8.5 ± 0.4 ps (ACN) and 1.0 ± 0.1 ps in PBS. The significantly rapid vibrational cooling lifetime (τ_2_) in water compared to that in acetonitrile is due to the ability of hydrogen bonds in the former solvent to promote rapid energy transfer from the hot solute to molecules in the first solvation shell.^19,20^ Importantly, as shown in Figure S4 and Table S2, vibrational cooling is known to be a wavelength-dependent process,^21^ and we are reporting herein an average lifetime for simplicity.

The proposed decay mechanism is further supported by the output of MD simulations. The time evolution of the states’ population is collected in Figure S13. Here, 89% of the trajectories were excited to the S_1_ state, identified as the lowest-lying ππ* state, and 11% to the dark (nπ*) state S_2_. The few trajectories starting on the S_2_ rapidly internally convert to the S_1_ (ca. 30–40 fs on average, Figure S13), revealing that the S_2_ (nπ*) does not play any significant role in the deactivation mechanism of 4OPy, contrary to what has been observed for uracil^22,23^ and other nucleobases.^24^ After 500 fs, most of the trajectories (73%) revert the population to S_0_, while a fraction of the population remains trapped in the excited state (27%). In agreement with the static calculations, all the trajectories starting in the S_1_ preserve the characteristic C2 puckered structure of the S_1_ minima, which is also maintained at the instant of the jump to the S_0_, coinciding with the geometries of the S_1_/S_0_ degeneracy points located quantum mechanically. Finally, intersystem crossing to the triplet manifold was found to be a residual route in vacuum (7%). The S_0_ population was adequately fitted using a Boltzmann sigmoidal function (Figure S14), which delivered an excited-state lifetime of 166 fs, of the same order of magnitude as the experimental τ_1_.

All the results reported above suggest that 4OPy should be equally photostable to UV radiation as other related canonical nucleobases. However, controlled low-intensity laser irradiation experiments at 267 nm in PBS and a careful monitoring of the trajectories reaching the S_0_ uncovered the formation of several photoproducts. In fact, the trajectories return to the S_0_ bifurcate between two different minima. About 77% of the trajectories reaching the S_0_ return to the original minimum, while 23% undergo the rupture of the C4–N3 bond, leading to a ketene product (see Scheme 1a).

**Scheme 1:**
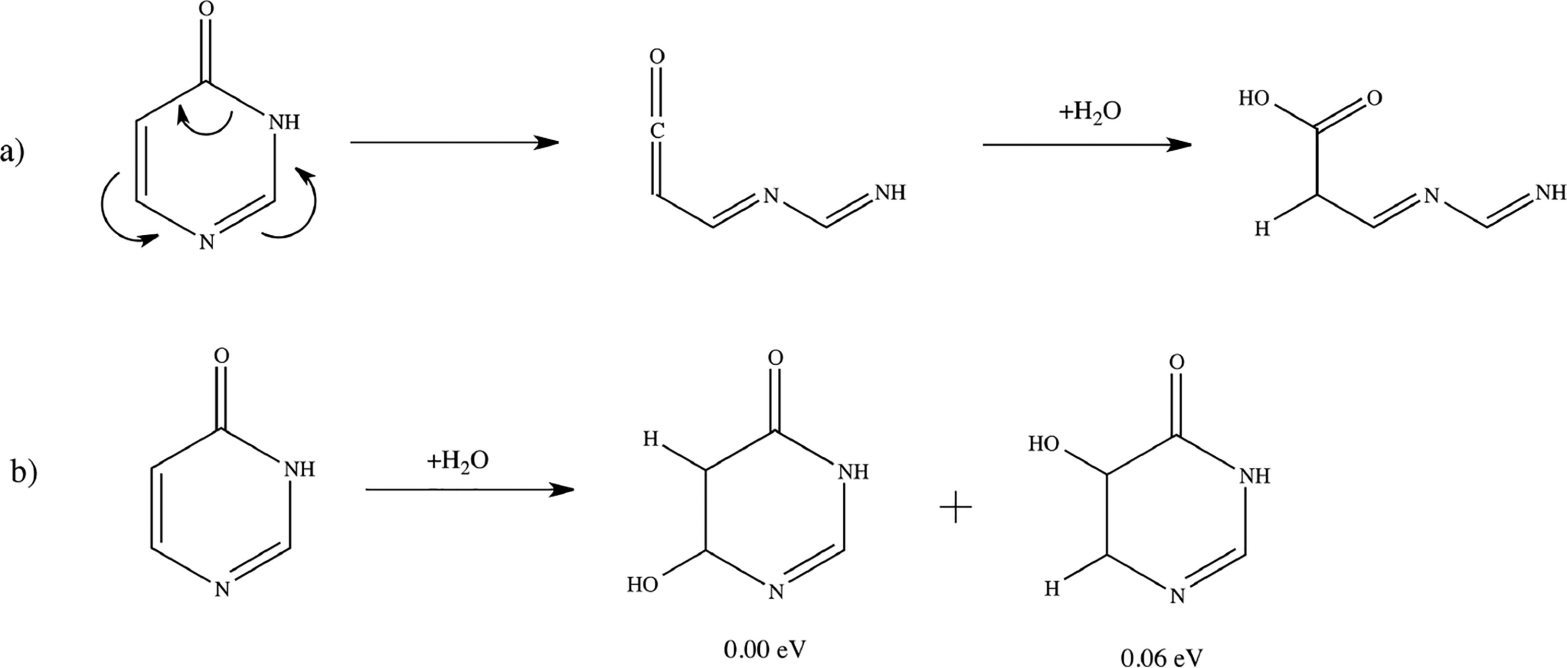
Photofragmentation Mechanism Observed in the MD Simulations Leading to the Ketene Product (middle) and the 3-(*N*-(Iminomethyl)imino)propanoic Acid (right) (a) from the Vibrationally-Excited S_0_ and 6-Hydroxy-5H-4-pyrimidinone (Predominant, 0.00 eV) and 5-Hydroxy-6H-4-pyrimidinone (0.06 eV) Photoproducts (b)

A minor (2–10%) C4–N3 ring-opening channel was previously observed in CASSCF MD simulations for uracil, but CASSCF is known to underestimate the energy of the dissociative conical intersections, reducing the importance of this process for uracil.^22,23^ It should be remarked that while the route leading to pyrimidine dissociation in 4OPy is mediated by the predominant funnel to the S_0_ (CI_S1/T1/S0_, Figure 3a), ring fragmentation takes place through an open-ring-crossing lying 0.5 eV above the main S_1_/S_0_ IC funnel in uracil.^22^ Interestingly, ketene in 4OPy is directly formed from CI_S1/S0/T1_ through a barrierless S_0_ profile, not requiring the return of the system to the original equilibrium S_0_ minimum (Figure 3b, red vs black curves). Moreover, our calculations reveal that the formation of the ketene is driven by dynamical effects, because for all the dissociative trajectories the momentum is accumulated along the C4–N3 bond (Figure S17).

From the experimental point of view, steady-state absorption spectra were obtained at selected irradiation times at 267 nm. As shown in Figures S7 and S8, chromophore loss is observed at the high-energy band maxima at ca. 220 nm in both solvents over a 10 min irradiation span, while an increase in the absorbance is detected at ca. 262 nm (20%) and at 315 nm (3%) in PBS (Figure S8). To further characterize the photochemistry of 4OPy in PBS, reverse phase high performance liquid chromatography (RP-HPLC) was used to separate the parent chromophore from its photoproducts following 267 nm irradiation. As shown in Figure 4a, the main elution peak at ca. 11 min corresponds to the 4OPy parent molecule. Elution peaks of lesser intensity were observed at times of ca. 6 and 16 min.Figure 4b records the absorption spectra of both primary photoproducts and of the parent molecule. Importantly, while it appears that the second eluted species is formed in larger quantity than the former, without knowing the molar absorption coefficients for each photoproduct, this cannot be definitively justified, as both concentration and the absorption cross section of each species will contribute to the intensity of the elution peak.

**Figure 4:**
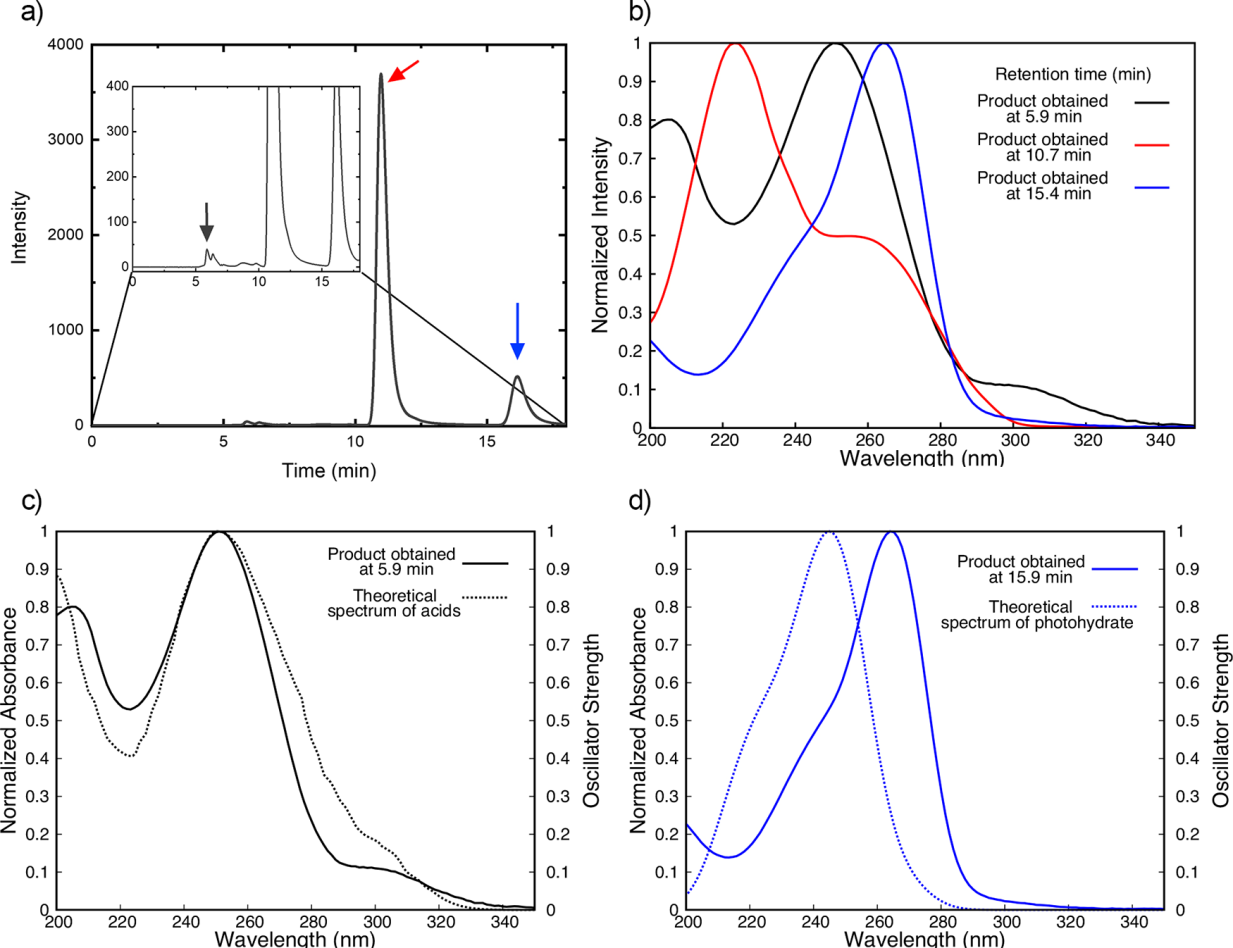
RP-HPLC chromatogram of 4OPy in ultrapure water following irradiation at 267 nm for 20 min (a). Absorption spectra of 4OPy (red), carboxylic acid (black), and 4OPy-hydrate (blue) (b). Superposition of the experimental (solid lines) and simulated (dotted lines) absorption spectra of the photoproducts (c and d).

Given the results from the MD simulations and considering the experimental conditions where the spectroscopic measurements were undertaken, we assign the early eluted compound to a product arising from hydrolysis of the unstable ketene intermediate, Figure 4c, resulting from the photodissociation of the C4–N3 bond: 3-(*N*-(iminomethyl)imino)propanoic acid (Scheme 1a). In fact, there is excellent agreement between the experimental absorption spectrum of this photoproduct and the simulated absorption obtained from the DFT MD simulations in the S_0_ of the three most stable conformers of the carboxylic acid in vacuum (see the Supporting Information for details).

The second photoproduct is assigned to the most stable 6-hydroxy-5H-4-pyrimidinone hydrate, Figure 4d, also observed for related canonical pyrimidine nucleobases when continuously irradiated with UV light.^25−30^ This product is the result of a nucleophilic hydrolysis reaction at the C5–C6 double bond in S_0_ (Scheme 1b). The S_0_ potential energy landscape for this reaction is illustrated in Figure 3c. According to our XMS-CASPT2 calculations, and similarly to uracil,^31,32^ 2.22 eV of energy is necessary to surmount the energy barrier separating the dispersively bound H_2_O-4OPy compound from the hydrate, which is well below the energy of the IC funnel. This demonstrates that the formation of this photoproduct occurs from the vibrationally hot S_0_, as previously suggested in other works.^32^ Also for this photoproduct, we obtain an excellent agreement between the experimental and the simulated absorption spectra resulting from an MD simulation of the hydrate (Figure 4d).

Through the powerful combination of time-resolved spectroscopy and molecular simulations, we have scrutinized the impact of including (removing) an oxo exocyclic group at the C4 (C2) position on the optical and photophysical properties of pyrimidine (uracil). Collectively, and in agreement with the effect of substitution in the equivalent C6 position in purines,^33^ we find that the incorporation of an oxo group in position C4 of pyrimidine (i) leads to a significant blue shift (ca. 0.4–0.7 eV) of the absorption spectrum, affecting to a larger extent the second absorption band; (ii) notably decreases the excited states lifetimes, which in 4OPy become from one to several orders of magnitude shorter than in pyrimidine; and (iii) greatly stabilizes the first ππ* excited state, producing a change in the excited state ordering and altering the nature of the spectroscopic state. This has important implications for the decay mechanisms of pyrimidine and 4OPy nucleobases, because the nπ* to ππ* ordering change of the lowest-lying excited state when moving from pyrimidine to 4OPy blocks the active singlet/triplet funnels dictating the photophysics of the pyrimidine core,^34^ concurrent with the activation of very efficient IC funnels to the S_0_ (similar to those found for uracil).^35^ We also conclude that ultrafast IC to the S_0_ does not guarantee the photochemical integrity of nucleobases, because the evolution of the systems in a vibrationally excited S_0_ can lead to the formation of photoproducts. This is particularly the case for photohydrates and ketene-derived photoproducts formed from the dissociation of the pyrimidine chromophore.

## Supporting Information Available

Experimental methods, materials, TAS and EADS following 290 nm irradiation, computational details, more extended analysis of the potential energy surfaces and dynamics simulations, and cartesian coordinates (PDF)
