# Supplementary material for: Disclosing the Role of C4-Oxo Substitution in the Photochemistry of DNA and RNA Pyrimidine Monomers: Formation of Photoproducts from the Vibrationally Excited Ground State
Source: J Phys Chem Lett. 2022 Feb 22;13(8):2000–6. doi: 10.1021/acs.jpclett.2c00052 (PMC8900130; doi:10.1021/acs.jpclett.2c00052)
Supplement: Supplementary file 1 — jz2c00052_si_001.pdf [file jz2c00052_si_001.pdf]

Supporting Information (39 pages)

Disclosing the Role of C4-Oxo Substitution in the Photochemistry of DNA and RNA Pyrimidine Monomers: Formation of Photoproducts from the Vibrationally Excited Ground State

Eva Vos,[a,†] *Sean J. Hoehn,*[b,†] *Sarah E. Krul,*[b,†] *Carlos E. Crespo-Hernández,*[b]* *Jesús González-Vázquez,*[a,c]* *Inés Corral*[a,c]*

[a] Departamento de Química, Módulo 13, Universidad Autónoma de Madrid, 28049, Madrid, Spain. [b]Department of Chemistry, Case Western Reserve University, 10900 Euclid Ave., Cleveland, Ohio 44106, United States. [c]Institute for Advanced Research in Chemistry (IAdChem), Universidad Autónoma de Madrid, 28049, Madrid, Spain. [†] these authors contributed equally to this work

Corresponding Author

* carlos.crespo@case.edu; jesus.gonzalezv@uam.es; ines.corral@uam.es

Table of Contents

Materials and experimental methods.	S-3
Transient absorption spectroscopy.	S-3
Reversed Phase – High Performance Liquid Chromatography (RP-HPLC).	S-3
Experimental TAS, EADS, global lifetimes and normalized kinetic traces.	S-4
Photodegradation study.	S-8
Computational details.	S-9
Vertical excitation energies. Gas phase absorption spectrum.	S-11
Triplet potential energy surface.	S-12
Geometries of selected stationary points.	S-14
Non-adiabatic molecular dynamics simulations.	S-15
Theoretical photodegradation study.	S-18
Theoretical transient absorption spectra.	S-20
Minimum energy paths.	S-25
Cartesian coordinates.	S-33

Materials and experimental methods.

4-pyrimidinone (> 98% purity) and acetonitrile (99.8% purity) were purchased from Sigma Aldrich and Fisher Scientific, respectively, and used as received. Phosphate buffer solutions with a total phosphate concentration of 16 mM from monosodium and disodium phosphate salts dissociated in ultrapure water (Milli-pore) were freshly prepared the day of each laser irradiation experiment. The pH of the solution was adjusted using 0.1 M solutions of NaOH to the desired pH of 7.4 (± 0.1 pH units).

Transient absorption spectroscopy

The experimental setup for the femtosecond broadband transient absorption spectroscopy (TAS) technique and the method of data analysis have been described in great detail previously.[1,2] Briefly, a Ti:sapphire oscillator (Vitesse, Coherent) was used to seed a regenerative amplifier (Libra-HE, Coherent) to produce 100 fs pulses, centered at 800 nm, with a 1 kHz repetition rate. An optical rail kit (FKE series, EKSMA optics) was used to generate the excitation pulse at 267 nm as previously described,[1] whereas an optical parametric amplifier (TOPAS, Quantronix/light conversion) was used to generate the 290 nm pump pulse via second harmonic sum frequency of the idler frequency. A 2 mm translating CaF_2 crystal was used to generate the while light continuum from 320 nm to 700 nm. Experimental pump-probe conditions were adjusted to eliminate two photon conditions as previously described in detail.[3]

The samples were freshly prepared the day of the laser experiment with absorbances matched at 1.0 in in both solutions at each excitation wavelengths. The homogeneity of the solutions in a 2 mm path length fused silica cell was maintained by continuous stirring with a Teflon-coated magnetic stirbar. The solution inside the cell was refreshed after every scan during data acquisition to minimize the potential contamination of the transient signals by the formation of photoproducts absorbing at the excitation wavelength.

Data collection made use of a home-made LabView program, following which global and target analysis were preformed using the Glotaran graphical user interface to the R-package TIMP software.[4] The complete multidimensional data was globally fit using a two component sequential kinetic model in ACN and a single exponential decay kinetic model in aqueous solution, convoluted with a Gaussian instrument response function of 250 ± 50 fs (FWHM). The evolution associated difference spectra (EADS) were extracted from the global and target analyses. Target analysis showing dependence of the single exponential decay lifetime on probe wavelength was performed using Igor Pro 6.37 (Wavemetrics, Inc.), convoluted with a Gaussian instrument response function of 250 ± 50 fs (FWHM).

Reversed Phase – High Performance Liquid Chromatography (RP-HPLC)

Samples were freshly prepared the day of each experiment in ultrapure water, pH 5.5, to have an absorbance of 1.0 at the excitation wavelength of 267 nm. Following one photon irradiation as described in the transient absorption spectroscopy methods, irradiated samples were analyzed by using HPLC (Shimadzu LC-20AD) associated with a photodiode array (PDA) detector (Shimadzu SPD-M20A) at room temperature. An Ascentis RP-Amide column (Supelco, 5 µm, 25 cm x 4.6 mm) was used to separate the

parent chromophore from its photoproducts. A 20 μL aliquot of the irradiated solution was injected into the HPLC through a manual injector. Ultrapure water (pH 5.5) was used to elute the sample with a flow rate of 0.5 mL min^{-1}.

Experimental TAS, EADS and normalized kinetic traces

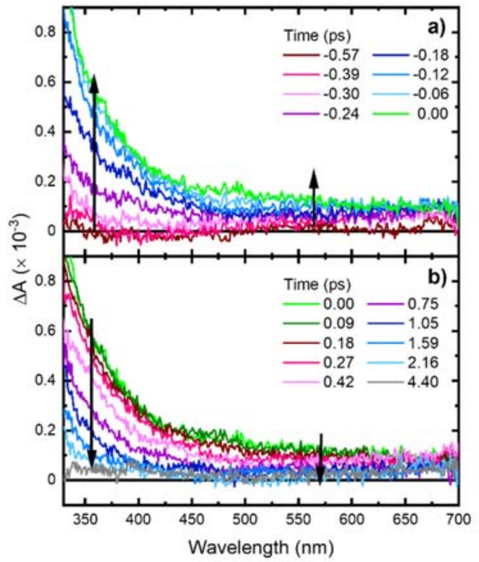

Figure S1. Transient absorption spectra of 4OPy in aqueous solution pH 7.4 following excitation at 267 nm.

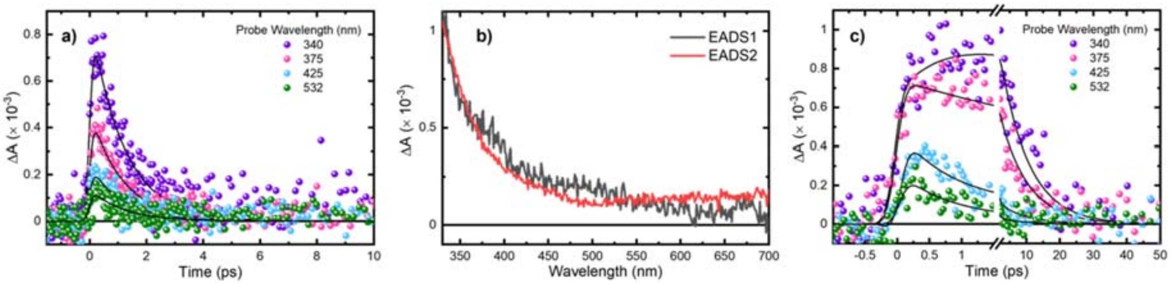

Figure S2. Representative traces (a) and evolution associated difference spectra (EADS) obtained from global and target analysis with a two-component sequential model in aqueous solution pH 7.4 (b) following excitation at 267 nm. Representative traces obtained from global and target analysis in ACN (c).

Table S1. Global average lifetimes of 4OPy in ACN and aqueous solution pH 7.4. All errors are reported as twice the standard deviation.

	ACN		Aqueous solution	
λ_{exc}	**267 nm**	**290 nm**	**267 nm**	**290 nm**
τ_1	0.8 ± 0.1 ps	1.0 ± 0.1 ps	< 0.25 ps	0.5 ± 0.1 ps
τ_2	8.4 ± 0.2 ps	8.5 ± 0.4 ps	1.2 ± 0.2 ps	1.0 ± 0.1 ps

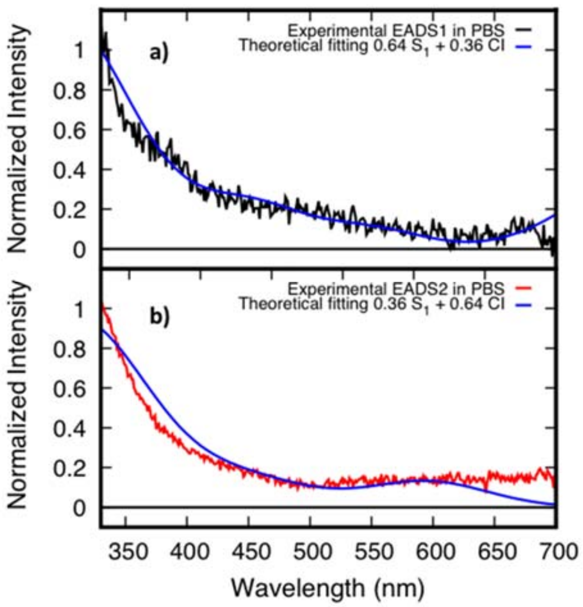

Figure S3. Theoretical fitting (blue line) over the experimental evolution associated difference spectra (black and red lines) obtained from global and target analysis with a two-component sequential model in aqueous solution pH 7.4 following irradiation at 267 nm.

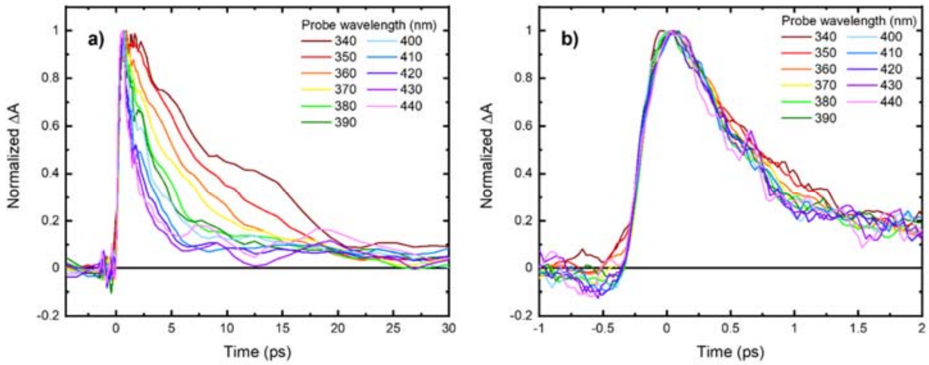

Figure S4. Normalized kinetic traces within the UV-near visible region (340-440 nm) of 4OPy following excitation at 267 nm in ACN (a) and aqueous solution pH 7.4 (b).

Table S2. Single exponential decay lifetimes of 4OPy in acetonitrile (ACN) and aqueous solution pH 7.4 following excitation at 267 nm. All errors are reported as twice the standard deviation.

	ACN	Aqueous solution
**Probe Wavelength**	**Lifetime (ps)**	**Lifetime (ps)**
340	9.36 ± 1.40	0.81 ± 0.11
360	6.16 ± 1.10	0.89 ± 0.10
380	4.49 ± 1.30	0.69 ± 0.09
400	4.39 ± 1.20	0.71 ± 0.10
420	0.78 ± 0.50	0.53 ± 0.10
440	0.35 ± 0.40	0.56 ± 0.09

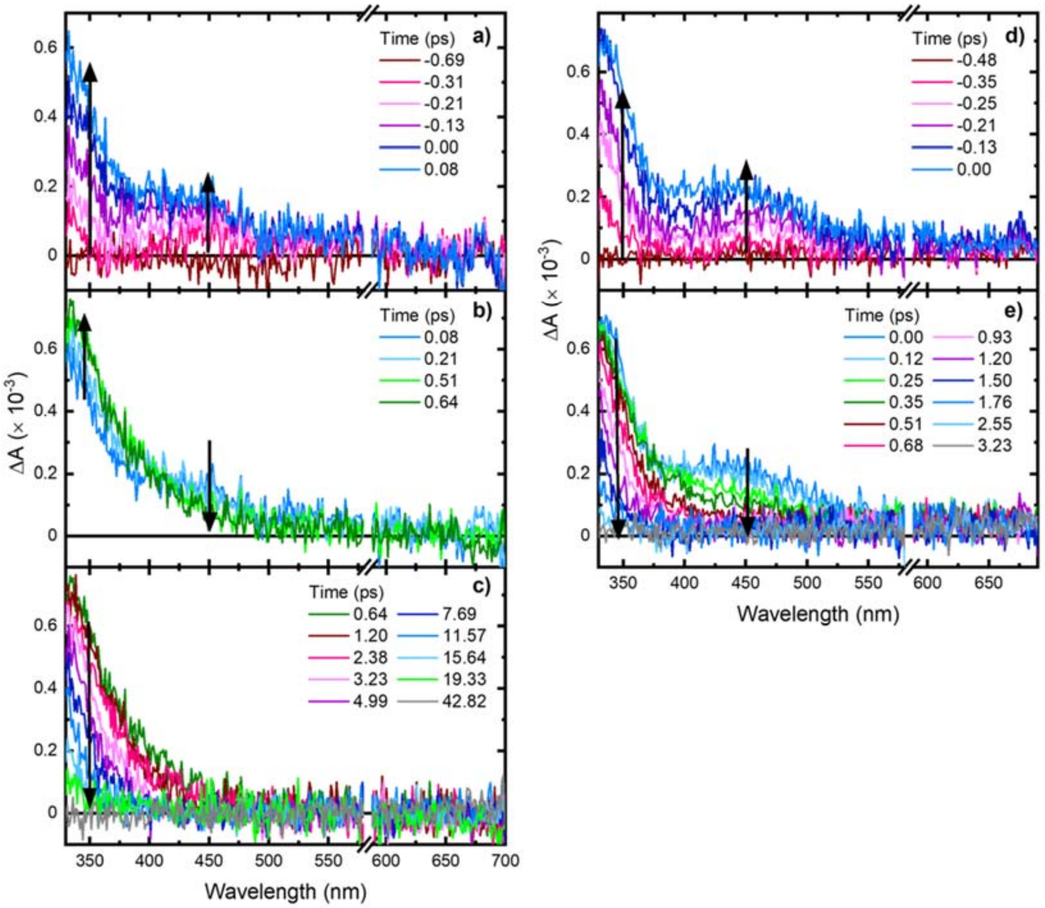

Figure S5. Transient absorption spectra of 4OPy in ACN (a-c) and phosphate buffer pH 7.4 (d-e) following excitation at 290 nm. Note the break in the abscissa to remove the overtone of the pump.

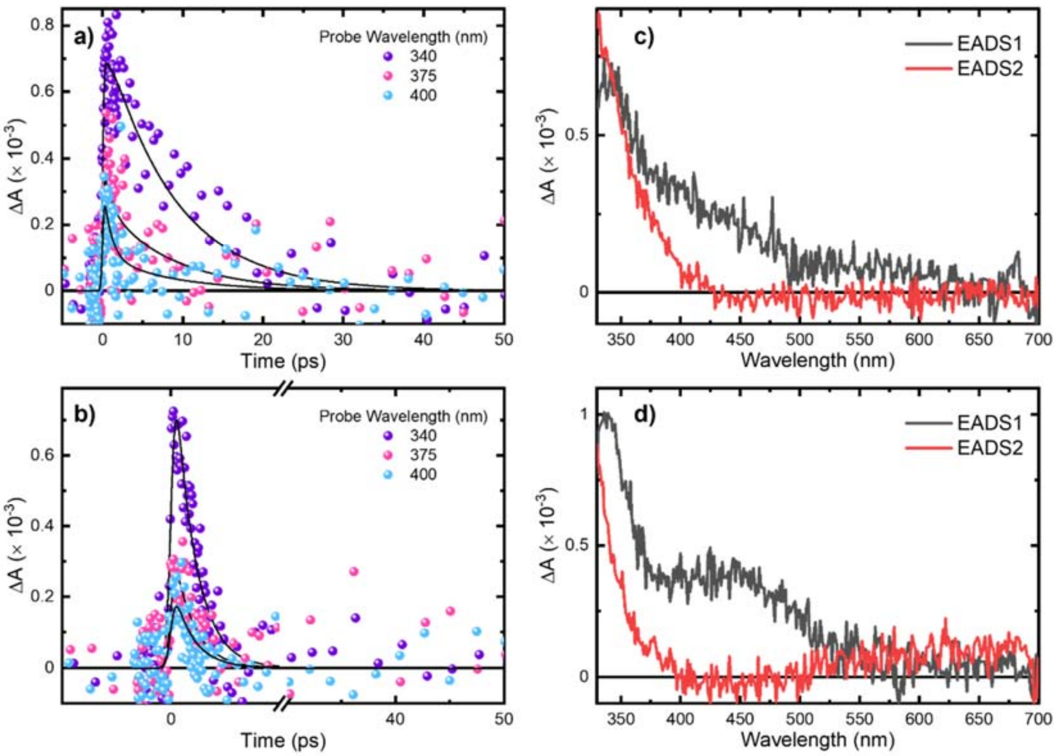

Figure S6. Representative traces (a-b) and evolution associated difference spectra (EADS) obtained from global and target analysis with a two-component model in acetonitrile (top) and aqueous solution pH 7.4 (bottom) following irradiation at 290 nm. Note a break in panel b the abscissa between 5 and 30 ps due to the transient species fully decaying in sub 5 ps in aqueous solution.

Photodegradation study

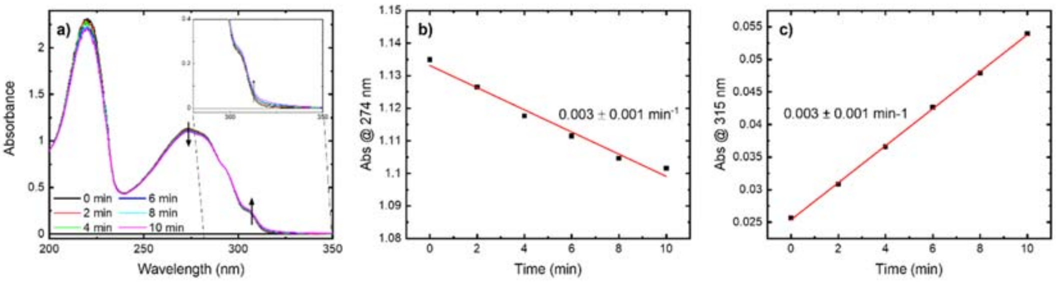

Figure S7. Controlled degradation of 4OPy in ACN following irradiation at 267 nm over 2-minute intervals for a total irradiation time of 10 minutes. Panel (b) displays the loss of absorbance at λ max of the lowest energy band (274 nm). Panel (c) displays the rise or increase of absorbance at 315 nm.

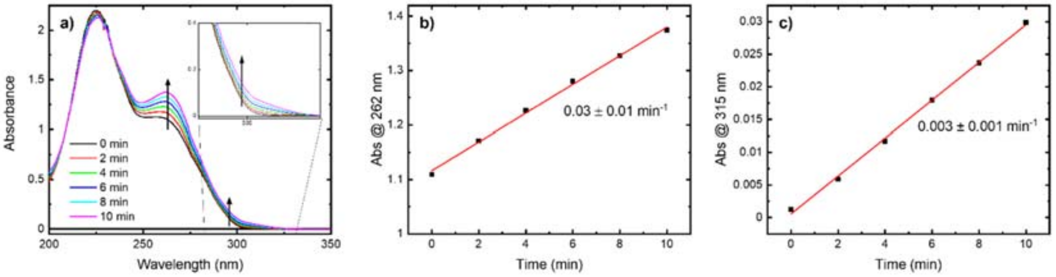

Figure S8. Controlled degradation of 4OPy in aqueous solution pH 7.4 following irradiation at 267 nm over 2-minute intervals for a total irradiation time of 10 minutes. Panel (b) displays the gain of absorbance at λ max of the lowest energy band (262 nm). Panel (c) displays the rise or increase of absorbance at 315 nm.

Table S3. Rates of degradation for 4OPy in ACN and aqueous solution (water) at the respective wavelength of the lowest energy band maximum (λ_{max}') and at 315 nm. Degradation plots at each respective lambda max and at 315 nm are shown in Figures S7 and S8.

	λ_{max}'	**315 nm**
**ACN**	0.003 ± 0.001 min^{-1}	0.003 ± 0.001 min^{-1}
**water**	0.03 ± 0.01 min^{-1}	0.003 ± 0.001 min^{-1}

Computational details

Previous to the theoretical study of the photophysics of 4-pyrimidinone (4OPy), we carried out a tautomer analysis in order to evaluate their relative stability. Altogether we considered four different tautomers, see Figure S9, whose stability was evaluated at the CCSD(T)[5]/aug-cc-pVTZ[6,7]/PCM(water)[8]//MP2/cc-pVTZ[9]/PCM(water) level of theory using ORCA 5.0.1 program.[10] According to our calculations and assuming a Boltzmann distribution at 298.15 K, 4-(3H)-pyrimidinone is the most stable tautomer and thus is expected to be the predominant compound with a relative population of almost 100%.

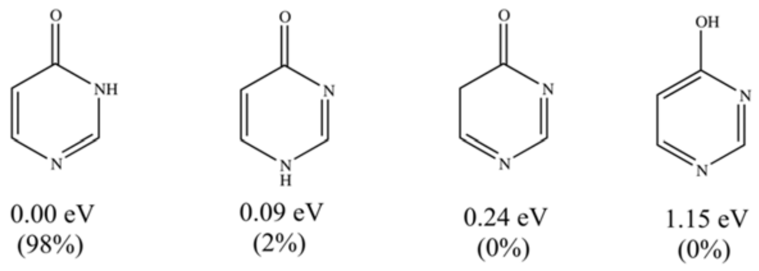

Figure S9. Relative energies in eV of 4OPy tautomers at CCSD(T)/aug-cc-pVTZ/PCM(water)//MP2/cc-pVTZ/PCM. Percentages in brackets indicate the relative population according to a Boltzmann distribution at 298.15 K.

Considering the results from the tautomer analysis, the photophysics of this system has been studied from a theoretical point of view considering the majority 4-(3H)-pyrimidinone tautomer.

For both the static and the dynamical study of 4OPy, the same protocol has been employed.

All the optimizations and gradient calculations have been performed with the extended multistate complete active space second order perturbation theory method, XMS-CASPT2,[11,12] as implemented in BAGEL program.[13] A second-order Douglas–Kroll–Hess Hamiltonian[14–16] has been employed in all the calculations, to account for relativistic effects. The ANO relativistic basis set contracted to triple-zeta quality (ANO-RCC-pVTZ)[6,17,18] and the cc-pVTZ-jkfit auxiliary basis set were used throughout. An imaginary level shift of 0.25 a.u. was employed to avoid the presence of intruder states and the IPEA shift was set to zero.

The active space employed in the multiconfigurational calculations is depicted in Figure S10. The (12,9) active space includes the complete π orbital system, plus two lone pairs respectively sitting at the N1 and the oxo substituent. The number of roots employed in the state average CASSCF calculations varies from calculation to calculation. For the modeling of the vertical absorption spectrum at the equilibrium ground state geometry (Table S4) and the simulated TAS (Figure S20-S26) we considered 10 singlet roots. The rest of the reported calculations were performed over 4 singlet and 4 triplet states.

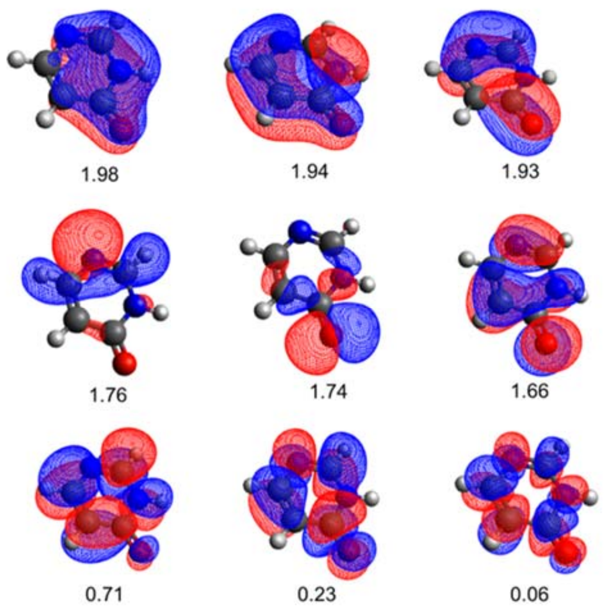

Figure S10. SA(4)-CASSCF/ANO-RCC-pVTZ orbitals with their occupation numbers included in the (12,9) active space employed in the multireference calculations at the ground-state equilibrium structure.

The main features of the potential energy landscape of 4OPy in the gas phase were explored resorting to the protocol described above. In particular, we have localized the minima, transition states and internal conversion funnels in the ground and in the singlet and triplet excited potential energy surfaces (PES) relevant to the deactivation of this system. The connection between these regions of the PES was ensured with the help of different theoretical tools, as linear interpolation calculations in internal coordinate (LIIC), minimum energy path calculations (MEP), or the nudged elastic band (NEB) method computed at the same level of theory described above. For the MEP calculations, a homemade interface between the ORCA program and BAGEL was used. This interface allows the coupling between the analytical XMS-CASPT2 gradients computed by BAGEL and ORCA's optimizer. Similarly, NEB calculations were performed combining BAGEL's XMS-CASPT2 gradients and the Atomic Simulation Environment (ASE) program[19] optimizer by a homemade interface.
The potential role of intersystem crossing in the deactivation mechanism of 4OPy was evaluated by locating intersystem crossing funnels along the main singlet deactivation routes. For the location of singlet-triplet crossings, we prepared a homemade code based on a penalty function.[20,21] The probability of population transfer at the ISC points was evaluated through the computation of the SOC with OpenMolcas.[22] For this purpose, the CI vector of the perturbatively modified CASSCF (determinant based) wave function was extracted from the BAGEL output and translated into OpenMolcas format (CSF based), to calculate the wave function overlap at different times and the SOC.

The time evolution of the excited system was monitored by means of singlet-triplet nonadiabatic mixed quantum-classical dynamics, considering the adiabatic representation. Within the trajectory surface hopping algorithm approach, nuclei are treated classically and follow Newton's equations, whereas electrons are treated quantum mechanically. For the integration of Newton's equations the velocity Verlet algorithm[23,24]

was employed with a time step of 0.5 fs. The evolution of the probability amplitudes determining the contribution of the different adiabatic states to the total wave function was integrated using the fourth order Runge–Kutta algorithm with a time step of 10^{-2} fs. A decoherence correction was applied, as recommended by Granucci and Persico with the parameters C = 1 and $\alpha = 0.1$ hartree.[25]
As already explained above, the electronic structure method employed for the molecular dynamics simulations was XMS-CASPT2(12,9)/ANO-RCC-pVTZ. For this, we combined BAGEL (to calculate the energy and the gradient of the spin-free states) and OpenMolcas codes (to evaluate the spin-orbit coupling and the overlap between the wave functions in two consecutive steps) with a homemade version of the molecular dynamics code SHARC[26] with a Surface Hopping based on the density[27] to follow the coupled electronic and nuclei movement.

The initial set of geometries and velocities employed for the modelling of the semiclassical spectrum and the dynamics was generated using a Wigner distribution for every normal mode of the electronic ground state, employing the harmonic MP2 frequencies.
From the original set of 1000 geometries, we simulated the semiclassical absorption spectrum shown in Figure 1 at the XMS-CASPT2(12,9)/ANO-RCC-pVTZ level of theory. To cut down the computational cost, in this case, the oscillator strengths were approximated considering PM-CASSCF wave functions. Gaussian functions with a width of 50 nm were employed to convolute the spectrum.
An energy window of 0.1 eV to both sides of the maximum of the calculated absorption band, i.e between 4.32 and 4.34 eV, was considered to select the initial pairs of geometries and velocities that were employed for the molecular dynamics of the system.
A total of 100 trajectories were run for a maximum time 500 fs. All the trajectories reaching the ground state were stopped once they had evolved either to the original heterocyclic minimum or to the ketene photoproduct.

Vertical excitation energies. Gas phase absorption spectrum.

Table S4 collects the gas phase vertical energies at the equilibrium geometry of 4-(3H)-pyrimidinone, characterized by a planar structure. According to our XMS-CASPT2 calculation using a SA10-CASSCF(12,9)/ANO-RCC-pVTZ reference, in the region comprised between 400 and 200 nm, there are two intense absorptions with similar oscillator strengths, the first (H $\rightarrow$ L) peaking at 287 nm (4.33 eV) and the second (H $\rightarrow$ L+1) at 224 nm (5.53 eV), both of $\pi\pi^*$ character, see orbitals in Figure S10. These two transitions are separated by two almost dark absorptions at 265 and 257 nm, corresponding to $n\pi^*$ states, and respectively involving the lone pairs of N1 and the carbonyl group, Figure S10. Table S4 also reports the computed permanent dipole moments of the electronic excited states evaluated at the S_0 minimum position, which, in the case of $\pi\pi^*$ states, exceed the value computed for the S_0.

Table S4: Vertical excitation energies and oscillator strengths for the first singlet and triplet states of 4-pyrimidinone in gas phase at XMS-CASPT2(12,9)/ANO-RCC-pVTZ//XMS-CASPT2(12,9)/ANO-RCC-pVTZ level of theory. The S_0 XMS-CASPT2 dipole moment is 2.468 D.

State	ΔE/eV (nm)	f	Permanent dipole moment (D)
S_1 (ππ*)	4.33 (287)	0.111	2.604
S_2 (nNπ*)	4.68 (265)	0.000	0.070
S_3 (nOπ*)	4.81 (257)	0.001	0.170
S_4 (ππ*)	5.53 (224)	0.175	2.889
S_5 (nNπ*)	5.57 (222)	0.000	0.089
S_6 (nOπ*)	6.28 (197)	0.006	0.493
T_1 (ππ*)	3.51 (353)		
T_2 (nπ*)	4.63 (268)		

Additional regions of the singlet potential energy surface.

Irradiation at the maximum of the first absorption band results in the partial population of the S_2(nπ*) excited state (11%). Thus, for completeness, we also mapped the potential energy surface along this electronic state. A MEP calculation along the S_2 was found to connect by a barrierless path the FC region with the S_2/S_1 crossing, where the T_2 state is also energetically degenerate. This crossing is directly connected with the S_1A(ππ*) minimum, as revealed by MEP calculations starting from the crossing position and following the state with ππ* character. A second MEP, this time following the nπ*state, describes a rather planar region of the PES (see Figure S11 and Figure S39) with nπ*character which latter evolves in a barrierless manner to the S_1A(ππ*) minimum. In fact, XMS-CASPT2 does not locate a nπ* minimum in the S_1 potential, even when significantly reducing the optimization step. This topography of the nπ* PES is fully consistent with the outcome of the MD simulations which predict an ultrafast decay of the S_2 population to the S_1.

Triplet potential energy surface.

Along the mapping of the PES of 4OPy, we also explored the topography of the triplet excited states. The energetic proximity of the triplet and singlet excited states at particular regions of the PES shown in Figure 3 suggests the possibility for population transfer. We find moderately coupled triplet states lying close to the position of the singlet minima, S_1A and S_1B (energy gaps <0.2 eV, SOC ca. 10-20 cm^{-1}). Indeed, the located S_1/S_0 internal conversion funnels actually correspond to $S_1/S_0/T_1$ three state degeneracy points, with the T_1 significantly coupled to the S_1 (SOCs 15-20 cm^{-1}) and the S_0 (SOCs 6-10 cm^{-1}). MEP calculations along the T_1 state starting both from the minima and the $S_1/S_0/T_1$ crossings lead to a T_1 minimum, T_{1min}, which preserves the ππ* character of the S_1 but not its characteristic puckered geometry, since the T_{1min} recovers the planarity of the ground state minimum.

Moreover, two higher lying additional S/T crossings were located, this time involving the second triplet state, T_2 (nπ*), (See Figure S11). The first crossing at 4.19 eV, which in fact coincides with the position of the S_2/S_1 conical intersection, shows a quite stretched CO bond distance and the oxo group slightly slanted in the direction of the N3 atom. The second S_1/T_2 crossing, 0.2 eV higher in energy than the former, presents as well a quite strained CO bond and a displacement of the C2HN3H moiety from the plane of the molecule. Minimum energy paths from these two crossings lead to a nπ* minimum in the T_2 potential, T_{2min}, which would internally convert to T_{1min} (ππ*) after accessing a T_2/T_1 crossing located ca. 0.3 eV above the T_{2min}. The spin-orbit coupling between S_1 and T_2 at the intersystem crossing regions is quite remarkable (ca. 25- 50 cm^{-1}) for a system consisting of atoms of the first period.

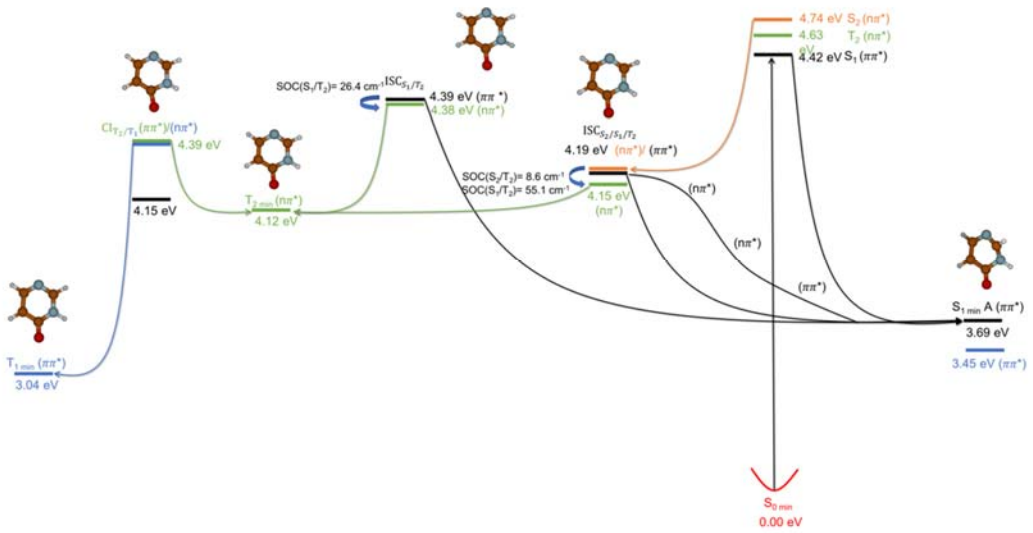

Figure S11. Scheme showing deactivation routes for 4-pyrimidinone involving the singlet nπ* state and the triplet manifold at XMS-CASPT2(12,9)/ANO-RCC-pVTZ level of theory.

Geometries of selected stationary points

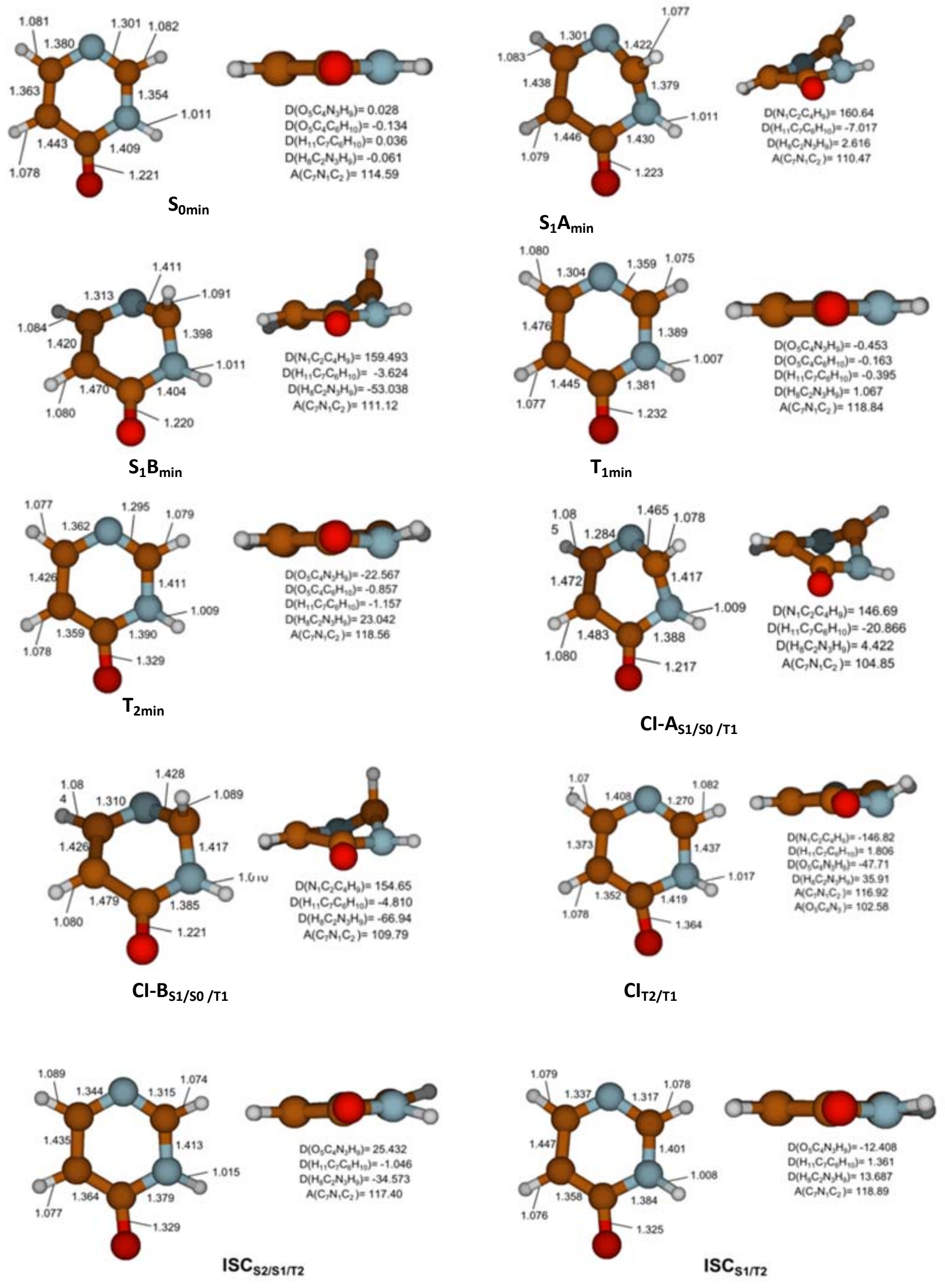

Figure S12. Representative distances (in Angstroms), angles and dihedrals (in degrees) for the XMS-CASPT2(12,9)/ANO-RCC-pVTZ optimized stationary points.

Non-adiabatic molecular dynamics simulations

The decay dynamics of 4OPy was investigated through the monitoring of 100 trajectories, propagated in the gas phase at XMS-CASPT2 level and employing a quantum classical surface hopping approach (see details above).

Consistent with the theoretical semiclassical spectrum (recall Figure 1), irradiation of the system within an energy window of 0.2 eV, centered around 4.34 eV (286 nm), would excite 89% of the trajectories to the S_1 state, identified as the lowest lying $\pi\pi^*$ in the vertical absorption spectrum, and 11% to the dark ($n\pi^*$) state S_2, also contributing in a lesser extent to the first absorption band of the system. After 500 fs, 73% of the trajectories revert to the S_0, whilst a fraction of the population remains trapped in the excited state (27%). From the population remaining in the excited state, 7% was found to undergo intersystem crossing to the triplet manifold. For most of the trajectories, population transfer from the singlet manifold to the T_2 is observed through the two ISC presented in Figure S11. Part of the population in T_2 decays to the T_1 potential within the time window of our simulations. It must be, however, noticed that the deactivation mechanism involving triplet excite states is practically insignificant and emphasized that these simulations were done in the gas phase.

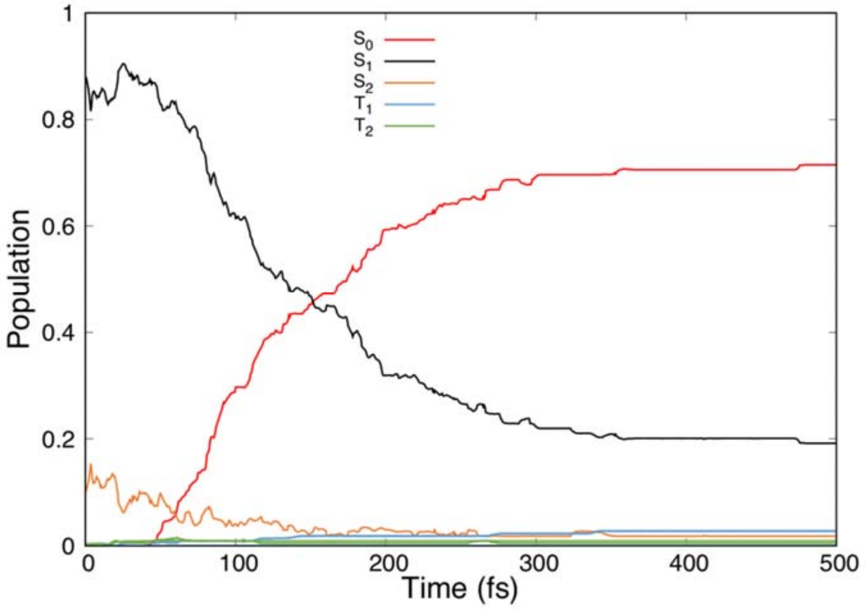

Figure S13: Time evolution of the average quantum probabilities of singlet and triplet states along the molecular dynamics simulations computed at XMS-CASPT2(12,9)/ANO-RCC-pVTZ level of theory.

The analysis of the trajectories decaying back to the ground state shows that more than 70% of the excited $^1\pi\pi^*$ population returns to the ground state in 500 fs, and ca. 35% does in the first 100 fs.

An approximated excited state lifetime can be extracted from the above results and compared with the experimental results. For this purpose, the total number of trajectories

in the ground state as a function of time have been fitted to a Boltzmann's sigmoidal function, see Figure S14.

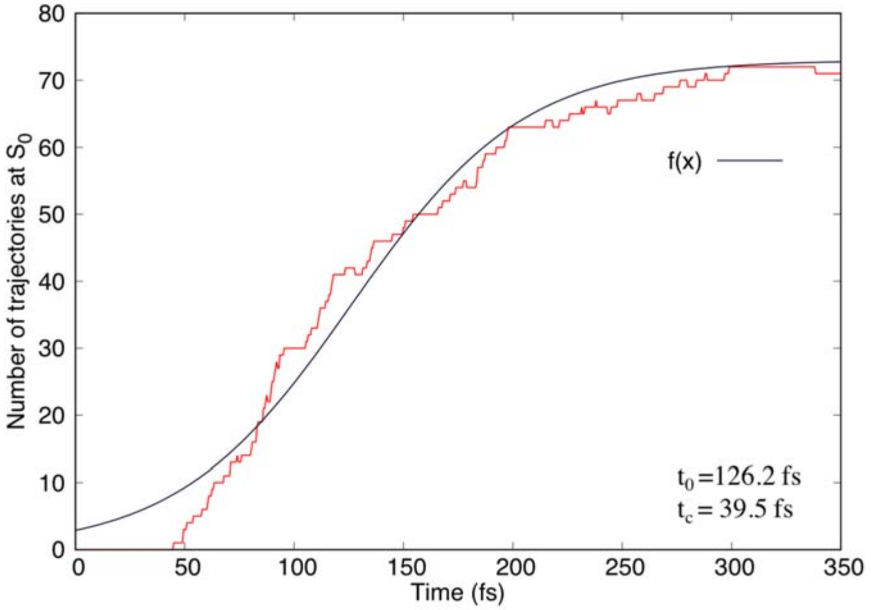

Figure S14: Fitting of the non-adiabatic molecular S_0 trajectories to a Boltzmann's sigmoidal function.

$$f(t) = \frac{1}{1 + e^{-\left(\frac{t-t_0}{t_c}\right)}} \qquad (1)$$

The fitting of the ground state population to the Boltzmann's sigmoidal function in (1) delivers an average lifetime (t_0) for 4OPy system of 126.2 fs. Due to the characteristics of the molecular dynamics approach used, not all the trajectories follow the same deactivation pathway, that is, some of them can get trapped in the minima for more time than others which are able to find a more favorable deactivation mechanism. For this reason, a deviation from this average lifetime of 39.5 fs has been estimated through a Boltzmann sigmoidal fitting, t_c. This function width indicates the possibility that the trajectories get retained in the potential energy landscape along the dynamics.

Representative trajectories

The trajectories shown in the following are representative of the two main deactivation routes observed for 4OPy which account for the recovery of the original ground state (Figure S15) and ketene formation (Figure S16).

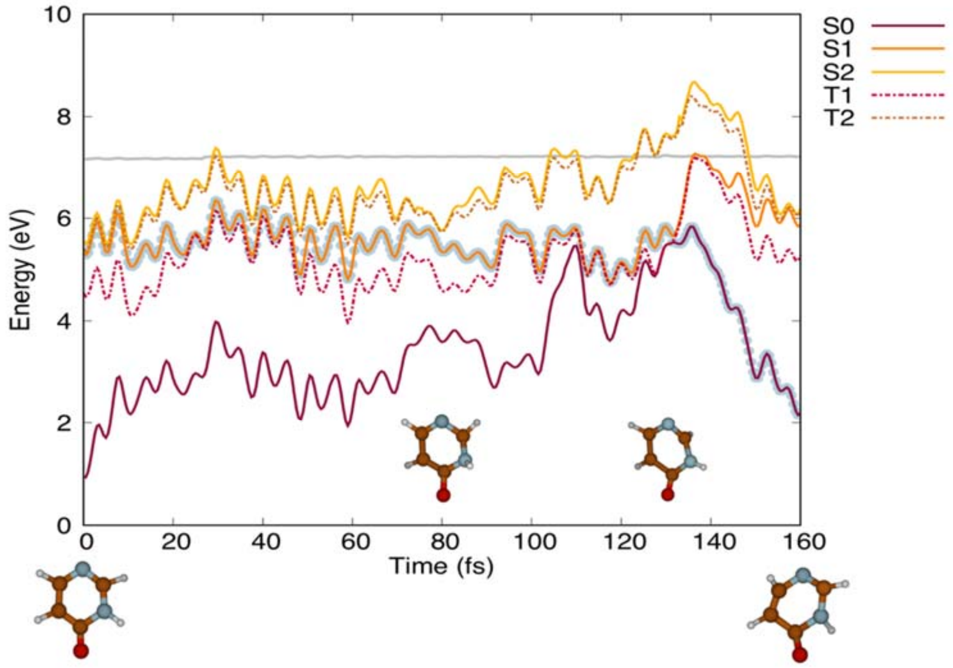

Figure S15: Representative trajectory for 4OPy returning to the original GS structure using XMS-CASPT2(12,9)/ANO-RCC-pVTZ as electronic structure method.

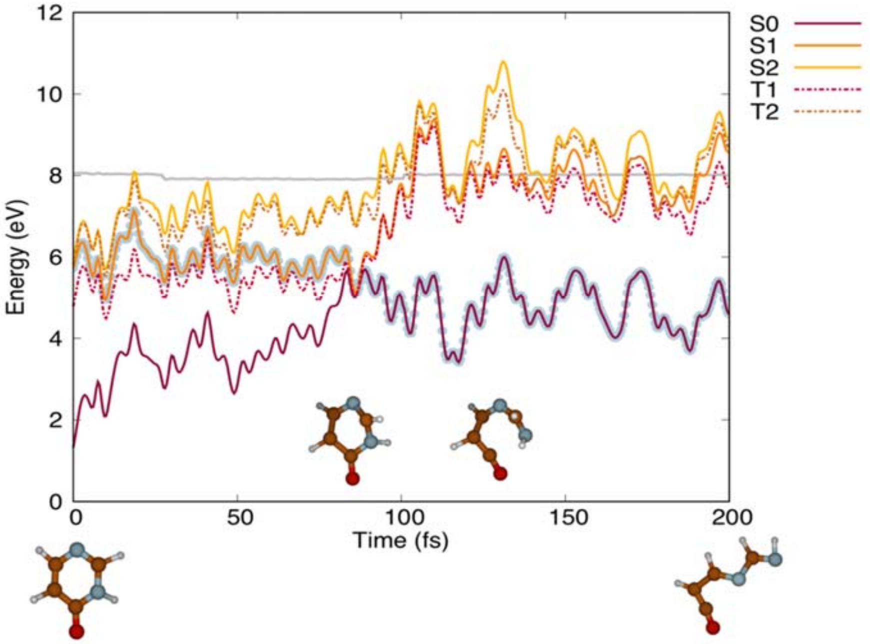

Figure S16: Representative trajectory of 4OPy leading to the ketene photoproduct using XMS-CASPT2(12,9)/ANO-RCC-pVTZ as electronic structure method.

Theoretical photodegradation study

NEB calculation between the CI-$B_{S1/S0/T1}$ and ketene

The C4-N3 bond breaking observed during the molecular dynamics simulations was also studied resorting to a Nudge Elastic Band (NEB) calculation interpolating the most stable optimized CI-$B_{S1/S0/T1}$ internal conversion funnel and the ketene open product, see Figure 3b. The first cycle of NEB (red line in Figure 3b) reveals that dissociation from CI-$B_{S1/S0/T1}$ point could take place directly, i.e. there is no barrier connecting these two points of the potential energy surface. The optimized reaction path (last cycle of the NEB calculation, black line in Figure 3b), however, would first drive the system to the original heterocyclic planar minimum, from where, after surmounting an energy barrier the system would access the global ketene minimum.
Interestingly, the NEB results reveal that the topography of the PES alone does not govern the pyrimidine ring-opening process and that the formation of this photoproduct is mainly a consequence of dynamical effects. In fact, the alignment of the momentum in the direction of the C4-N3 bond (see Figure S17) would corroborate that the ketene is rather a dynamically driven photoproduct.

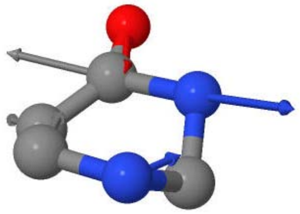

Figure S17: Momentum aligned in the direction of the C4-N3 bond collected from a selected trajectory leading to the ketene photoproduct.

Photoproducts spectra simulation

With the objective of identifying the photoproducts detected in the experimental sample after the 10 min irradiation, a computational spectroscopic study of several plausible photoproducts was carried out.

In order to monitor the evolution of the unstable ketene to the final photoproduct(s) detected in the experimental absorption spectra and due to the considerable flexibility of this intermediate, we selected one of the trajectories leading to the ketene species and reinitiated its propagation. The adiabatic dynamics for this trajectory in the ground state was monitored for 2 ps at the more computationally affordable B3LYP/cc-pVDZ level of theory using the RIJCOSX approximation.[28,29] This dynamics was employed to identify ketene most stable conformers. For that purpose, we recorded altogether 50 snapshots separated by a 40 fs time lapse. Then, these structures were reoptimized at B3LYP/cc-pVDZ level of theory leading to 4 different conformers. Assuming that the most probable fate of the ketene species is the attack of a water molecule and the formation of a carboxylic acid, we reoptimized the structures of the corresponding acids this time at the B3LYP/cc-pVDZ level of theory, obtaining the 3 conformers collected in Figure S18.

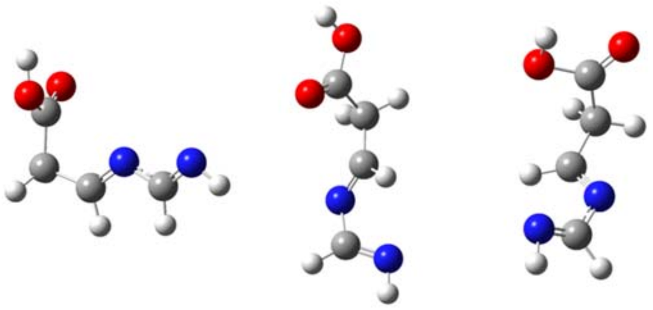

Figure S18. Three conformers of the carboxylic acid generated from the hydrolysis of the ketene photoproduct calculated at the B3LYP/cc-pVDZ.

For each of these conformers, we subsequently run ground state molecular dynamics simulations at the B3LYP[30]/aug-cc-pVDZ level, using the RIJCOSX approximation[28,29] and a NHC thermostat[31] initialized at 350 K during 500 fs considering a timestep of 0.5 fs. The absorption spectrum for each conformer was simulated considering the XMS-CASPT2(12,9)/ANO-RCC-pVTZ vertical excitation energies on the geometries registered every 5 fs from the DFT molecular dynamics. As for the semiclassical absorption spectra, the oscillator strengths were computed considering PM-CASSCF wave functions. Finally, the computed absorption spectrum for the carboxylic acid in Figure 4c was prepared considering the same population for the three tautomers.

The same procedure was used for the simulation of the absorption spectra of the 4OPy hydrates. In this particular case, however, the DFT molecular dynamics were directly initialized from the optimized hydrated species. In our simulations, we considered both the 5-hydro-6-hydroxy and the 5-hydroxy-6-hydro species although similarly to previous studies on uracil the former species was found to be more stable by 1.4 kcalmol^{-1}.[32] For the hydrates, a new active space (14,10) in Figure S19, has been considered to include the water orbitals involved in the hydration reaction.

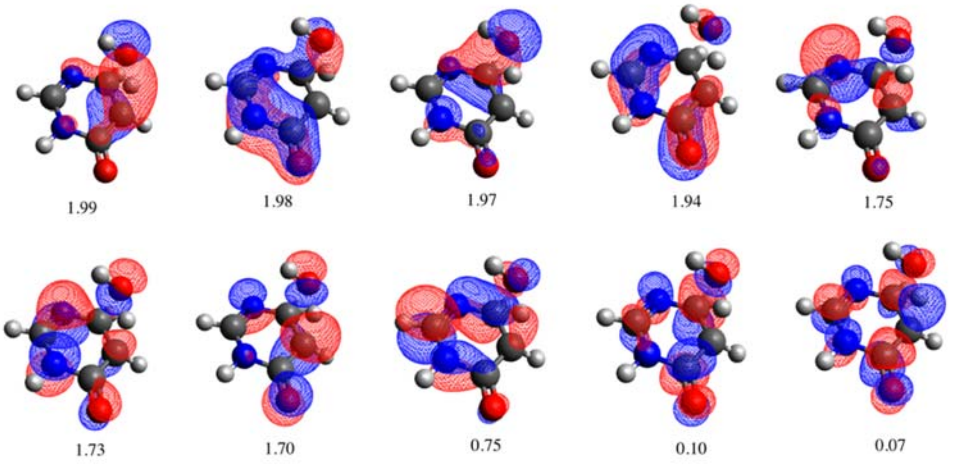

Figure S19. SA4-CASSCF/ANO-RCC-pVTZ orbitals with their occupation numbers included in the (14,10) active space employed in the multireference calculations of the 5-hydro-6-hydroxy 4OPy-hydrate. An equivalent active space was employed for the 5-hydroxy-6-hydro isomer.

The active space in Figure S19 was also employed for the photohydration potential energy profile in Figure 3c.

Theoretical transient absorption spectra

To help in the interpretation of the experimental transient absorption spectra (Figures 2a-c, S1 and S5) and evolution associated difference spectra (Figures 2d, and S2), we have computed the transient absorption at key regions of the excited potential energy surface of Figure 3a and S11, where the system is expected to spend some time along the deactivation mechanism. In particular, we have computed the vertical absorption energies and oscillator strengths at the singlet (S_1A_{min}, S_1B_{min}) and triplet (T_{1min}, T_{2min}) minima, and at the position of the $CI_{S1/S0/T1}$ conical intersections (CI-$A_{S1/S0/T1}$ and CI-$B_{S1/S0/T1}$) as a first approximation to the geometries for the vibrationally hot S_0 once internal conversion has occurred.

From the vertical excitation energies and the corresponding oscillator strengths at the position of each of the stationary points of the PESs, we have generated the individual theoretical transient absorption spectra reported in Figure S20 and Figures S21-S26. As already mentioned in the Computational details section, the simulated TAS have been computed at XMS-CASPT2(12,9)/ANO-RCC-pVTZ level of theory, including 10 singlet roots in the calculations.

The individual TAS were plotted using Gabedit 2.4.8 program[33], which assigns a Gaussian function to each vertical excitation. A full width at half maximum of 50 nm was selected for all the spectra. The simulated evolution associated spectra (EADS) replica in Figures 2e-f and S3 were constructed by linearly combining the individual transient absorption spectra to deliver the best possible experimental spectra, using a homemade program.

Figure S20 reveals that minimum S_1A_{min} shows a moderate absorption in the region between 600-400 nm and a strong absorption centered at 310 nm. On the contrary, the absorption signal of S_1B_{min} is moderate and spreads all along the region between 700 and 300 nm. Similar to S_1A_{min}, the absorption signal corresponding to the T_1 minimum peaks at 300 and 550 nm, but the singlet signal at 450 nm is replaced by another absorption above 600 nm. The T_2 instead shows a very weak absorption in this energy range, and thus it is not possible to monitor its population through the recording of transient absorption spectra. Both S_1/S_0 conical intersections, CI-$A_{S1/S0/T1}$ and CI-$B_{S1/S0/T1}$, show an absorption signal that follows an exponential decay within the considered energy range, presenting maximum around 300-350 nm and almost no absorption beyond 600 nm.

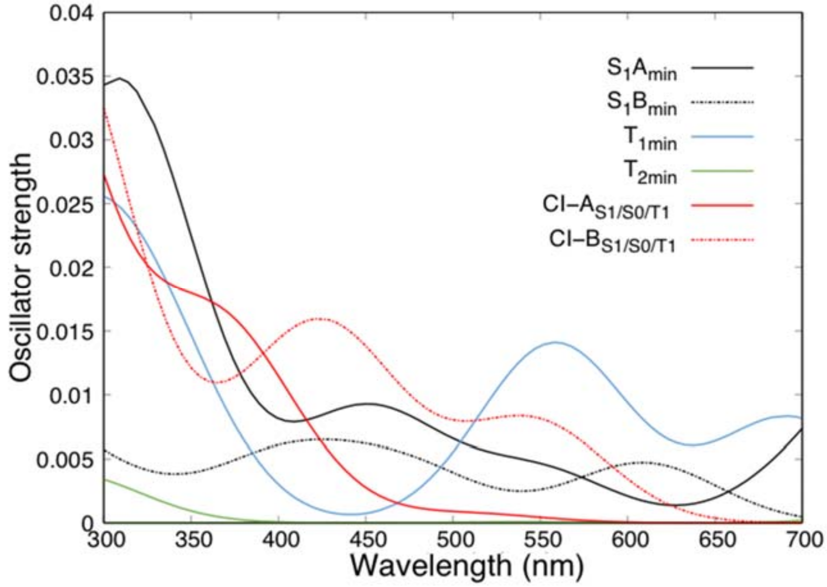

Figure S20: XMS-CASPT2(12,9)/ANO-RCC-pVTZ individual transient absorption spectra for the S_1A_{min} (black solid line), S_1B_{min} (black dashed line), T_1 (blue solid line), T_{2min} (green solid line), CI-$A_{S1/S0/T1}$ (red solid line) and CI-$B_{S1/S0/T1}$ (red dashed line) of 4OPy.

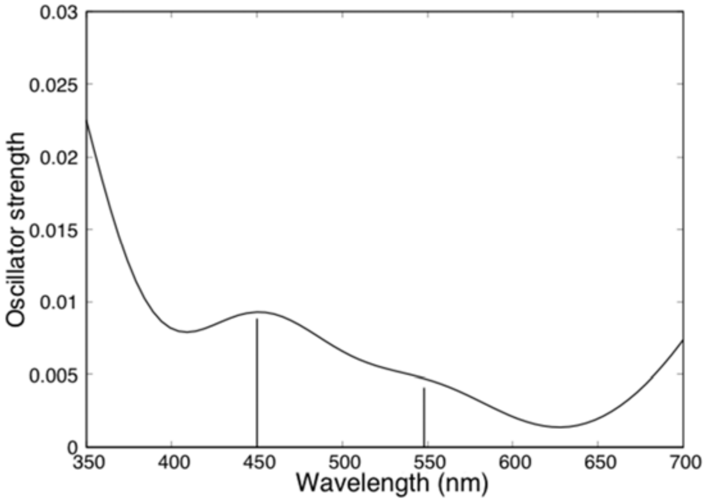

Figure S21. Transient absorption spectrum for S_1A_{min} minimum calculated at XMS-CASPT2/ANO-RCC-pVTZ level of theory.

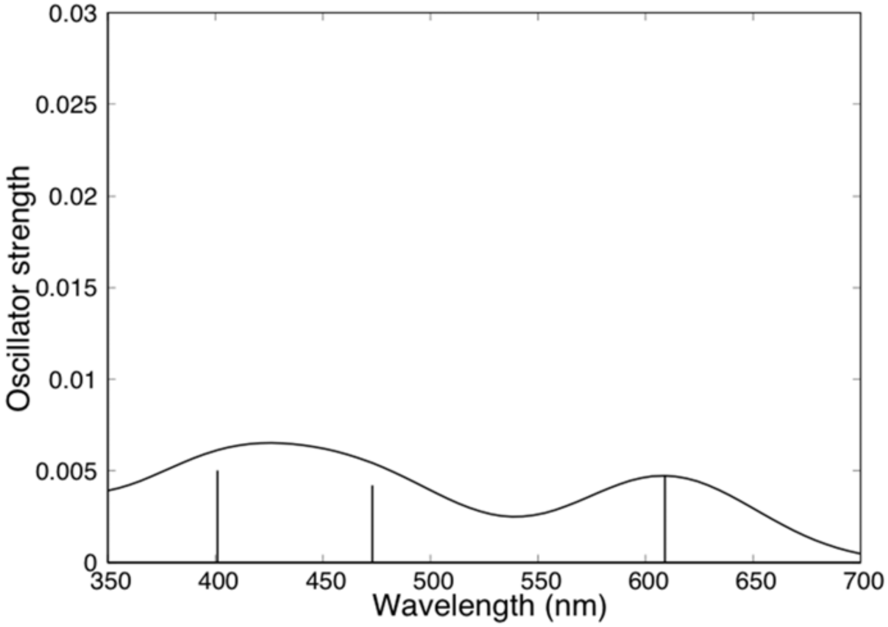

Figure S22. Transient absorption spectrum for S_1B_{min} minimum calculated at XMS-CASPT2/ANO-RCC-pVTZ level of theory.

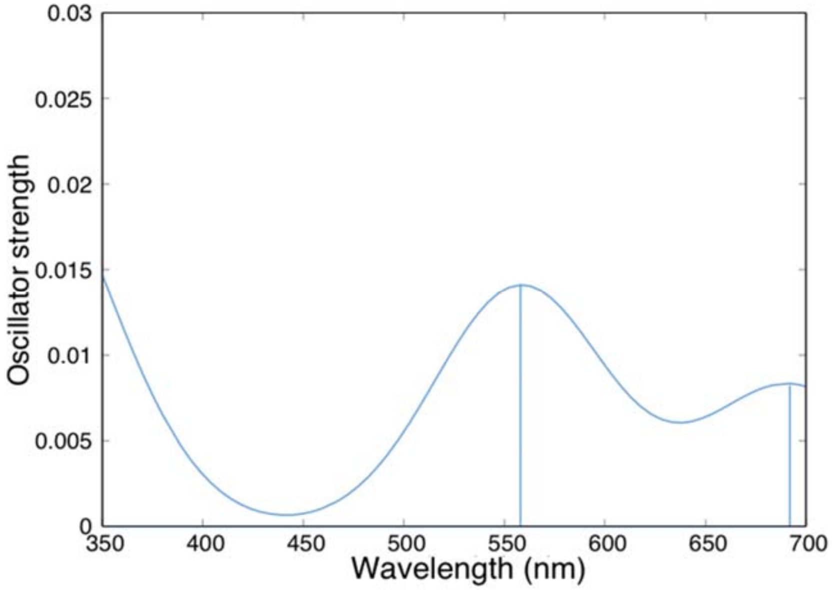

Figure S23. Transient absorption spectrum for T_{1min} calculated at XMS-CASPT2/ANO-RCC-pVTZ level of theory.

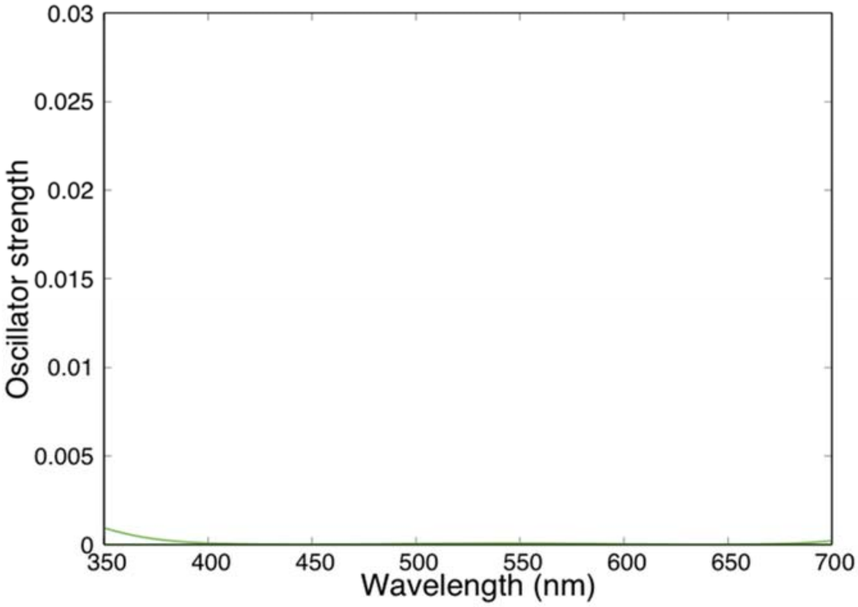

Figure S24. Transient absorption spectrum for T_{2min} calculated at XMS-CASPT2/ANO-RCC-pVTZ level of theory.

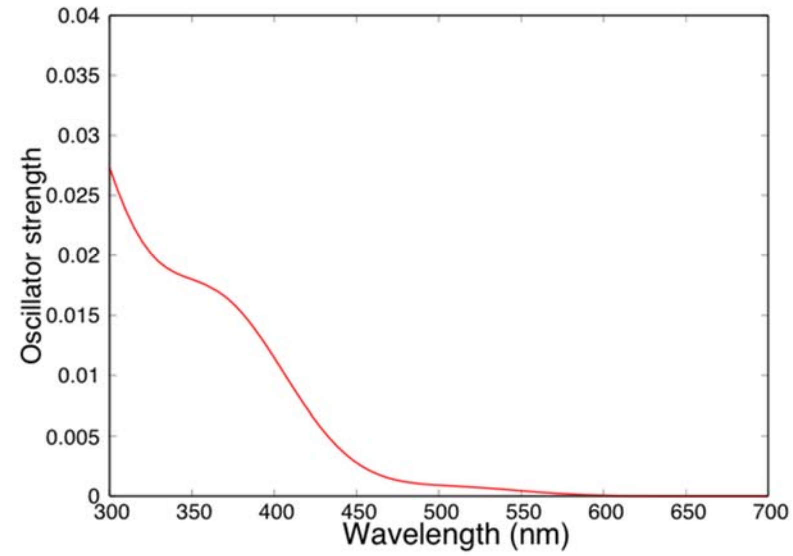

Figure S25. Transient absorption spectrum for CI-A$_{S0/S1/T1}$ conical intersection calculated at XMS-CASPT2/ANO-RCC-pVTZ level of theory.

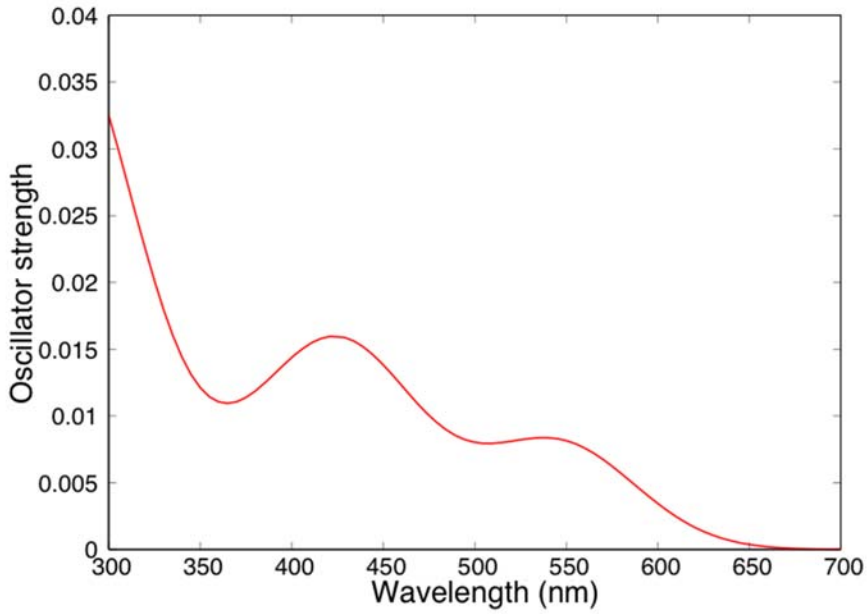

Figure S26. Transient absorption spectrum for CI-B$_{S0/S1/T1}$ conical intersection calculated at XMS-CASPT2/ANO-RCC-pVTZ level of theory.

Minimum energy paths

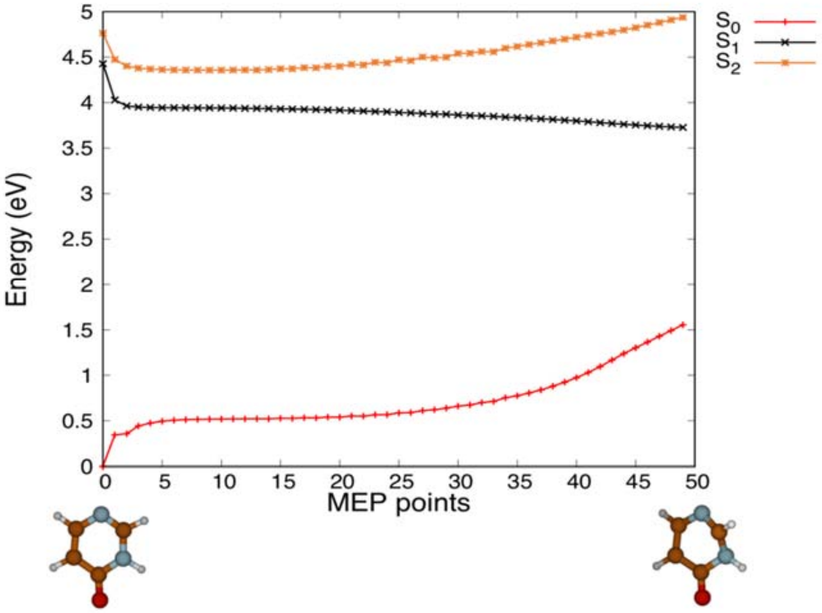

Figure S27. Minimum energy path from the Franck-Condon region along $S_1(\pi\pi^*)$ excited state at XMS-CASPT2(12,9)/ANO-RCC-pVTZ level of theory.

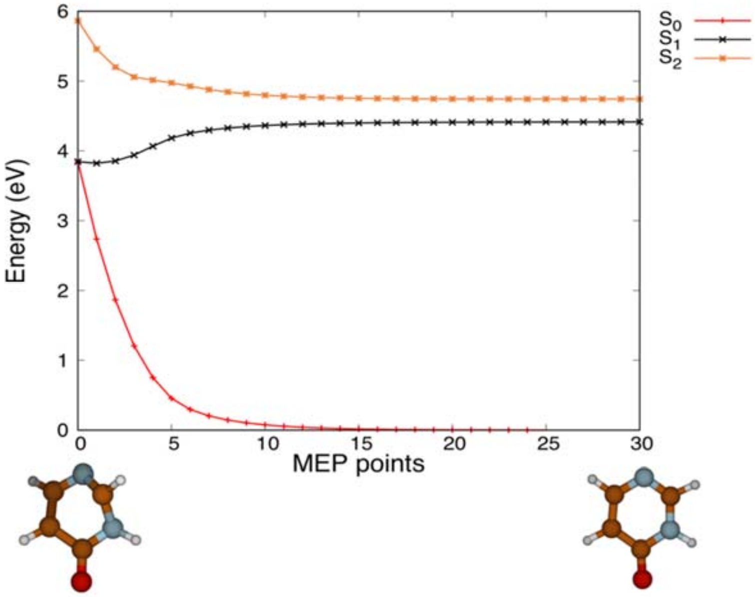

Figure S28. Minimum energy path from the CI-$A_{S1/S0/T1}$ along the S_0 state at XMS-CASPT2(12,9)/ANO-RCC-pVTZ level of theory.

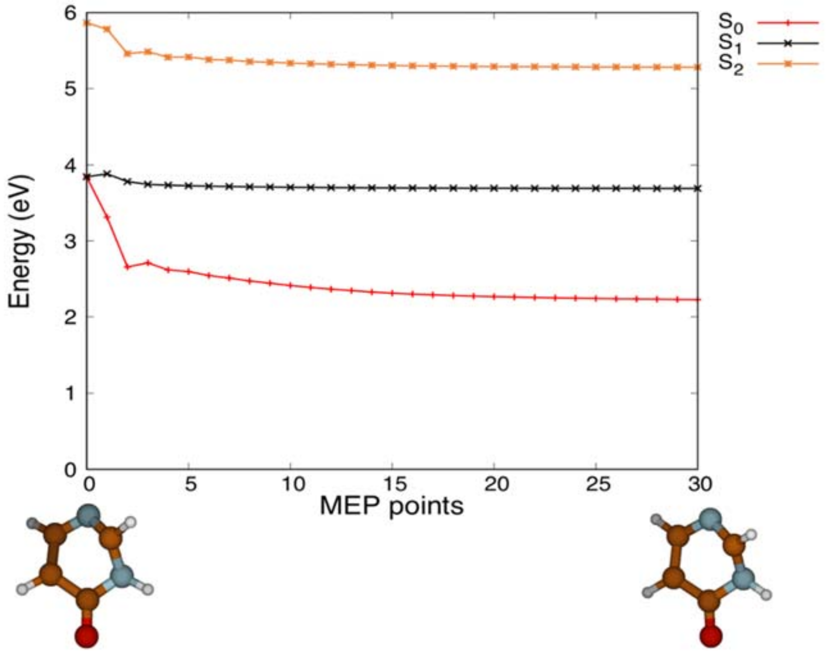

Figure S29. Minimum energy path from the CI-$A_{S1/S0/T1}$ along the $S_1(\pi\pi^*)$ excited state at XMS-CASPT2(12,9)/ANO-RCC-pVTZ level of theory.

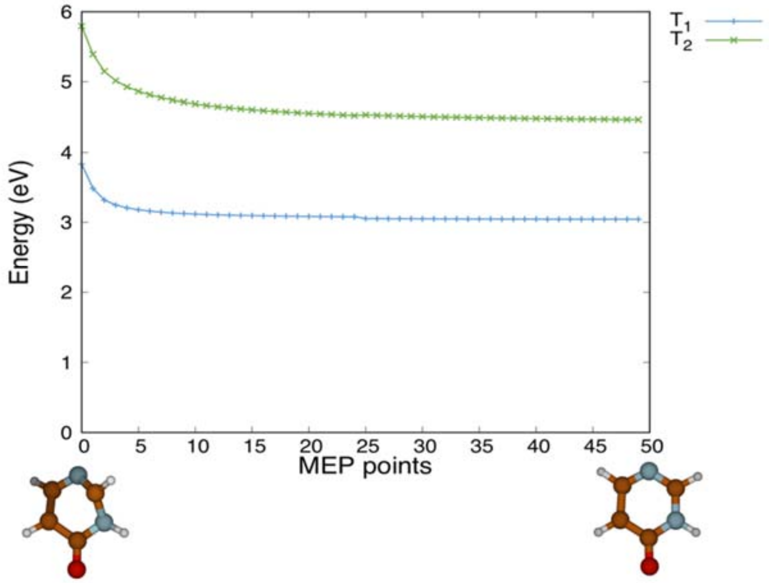

Figure S30. Minimum energy path from the CI-$A_{S1/S0/T1}$ along the T_1 excited state at XMS-CASPT2(12,9)/ANO-RCC-pVTZ level of theory.

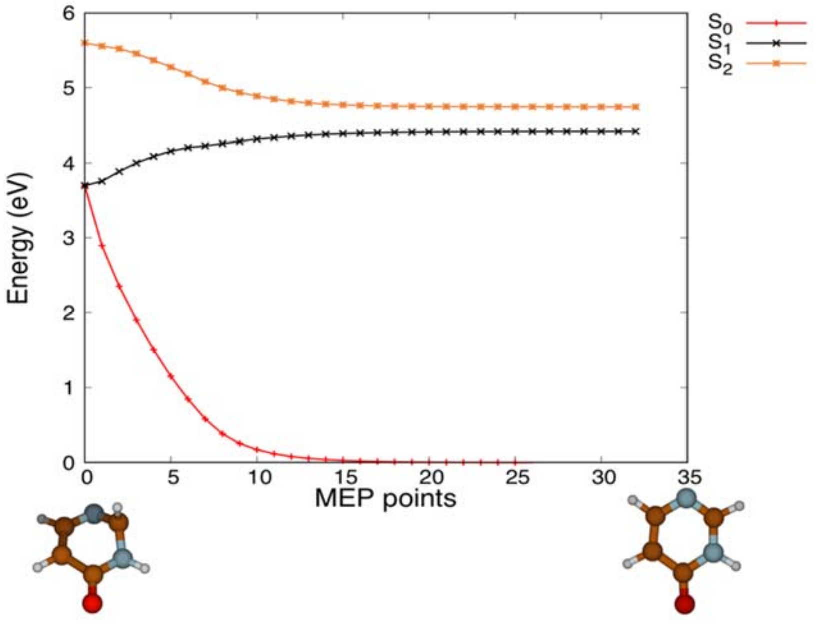

Figure S31. Minimum energy path from the CI-B$_{S1/S0/T1}$ along the S_0 state at XMS-CASPT2(12,9)/ANO-RCC-pVTZ level of theory.

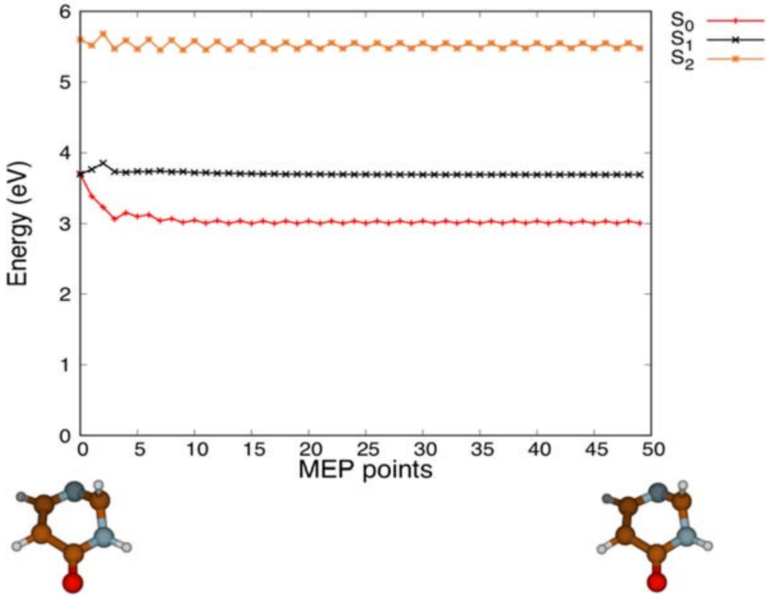

Figure S32. Minimum energy path from the CI-B$_{S1/S0/T1}$ along the $S_1(\pi\pi^*)$ excited state at XMS-CASPT2(12,9)/ANO-RCC-pVTZ level of theory.

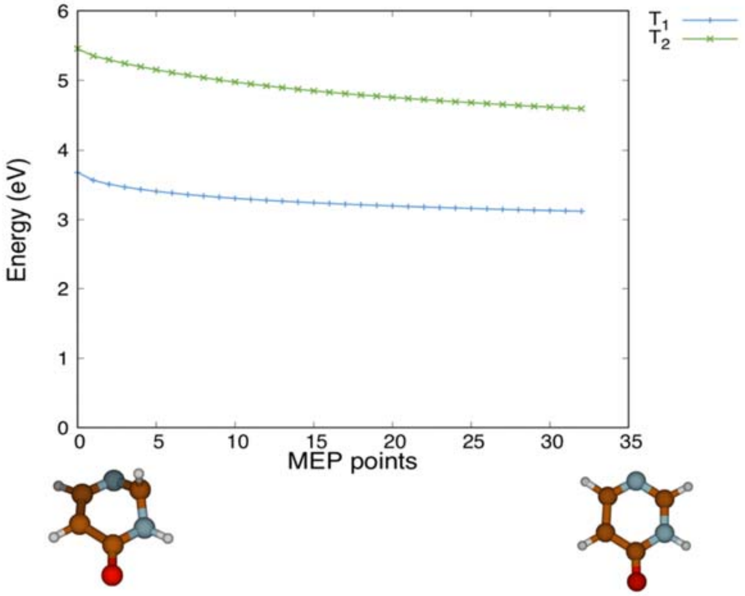

Figure S33. Minimum energy path from the CI-B$_{S1/S0/T1}$ along the T_1 excited state at XMS-CASPT2(12,9)/ANO-RCC-pVTZ level of theory.

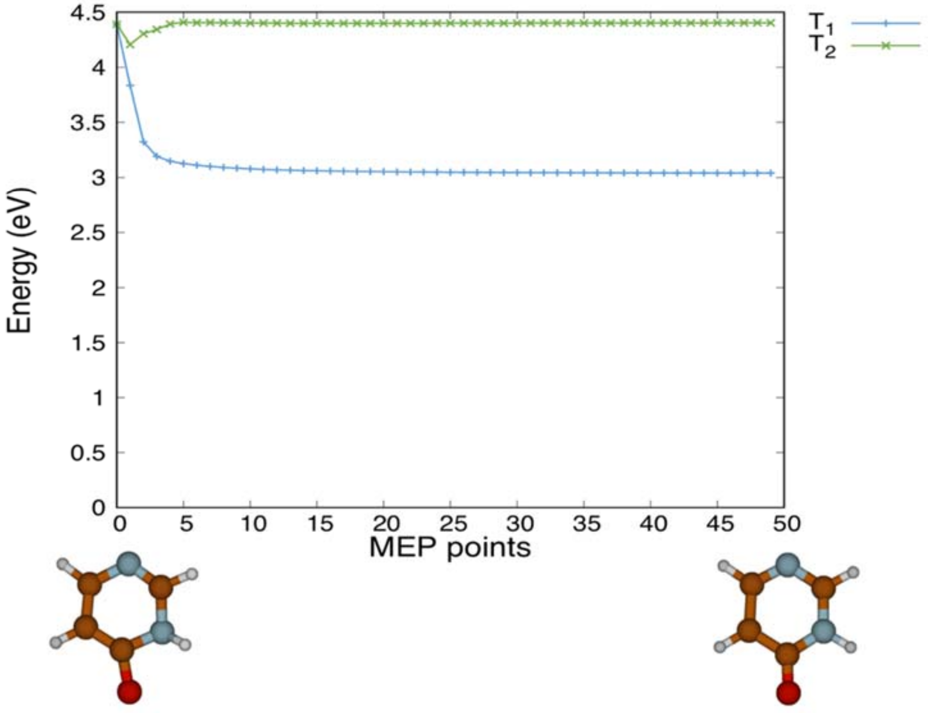

Figure S34. Minimum energy path from the CI$_{T2/T1}$ along the T_1 excited state at XMS-CASPT2(12,9)/ANO-RCC-pVTZ level of theory.

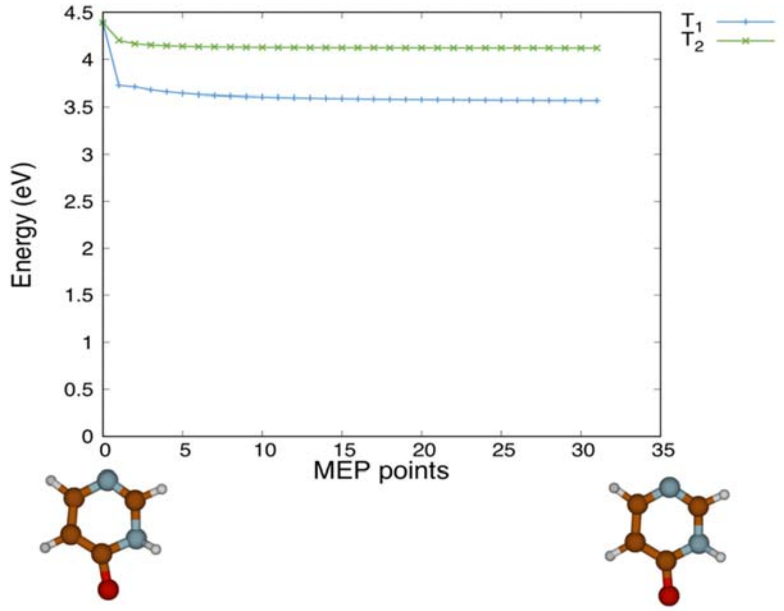

Figure S35. Minimum energy path from the $CI_{T2/T1}$ along the T_2 excited state at XMS-CASPT2(12,9)/ANO-RCC-pVTZ level of theory.

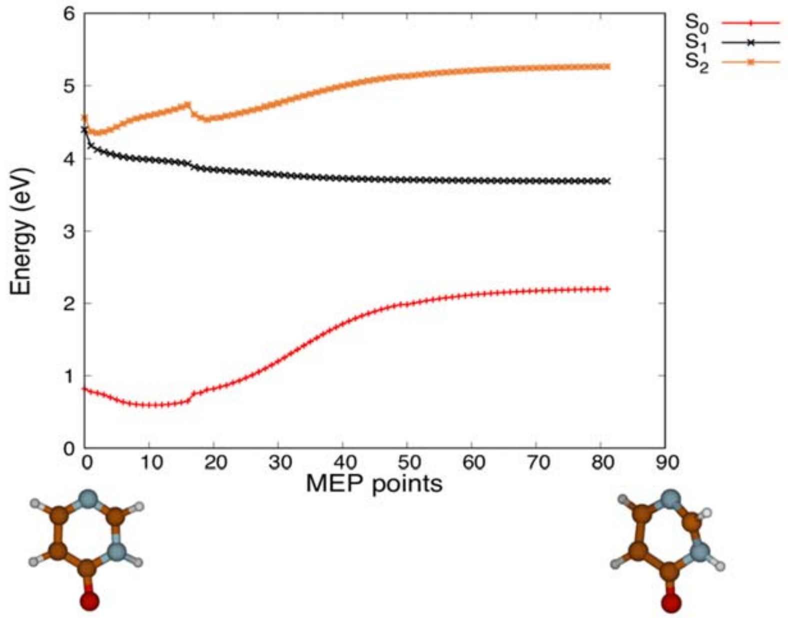

Figure S36. Minimum energy path from the $ISC_{S1/T2}$ along the $S_1(\pi\pi^*)$ excited state at XMS-CASPT2(12,9)/ANO-RCC-pVTZ level of theory.

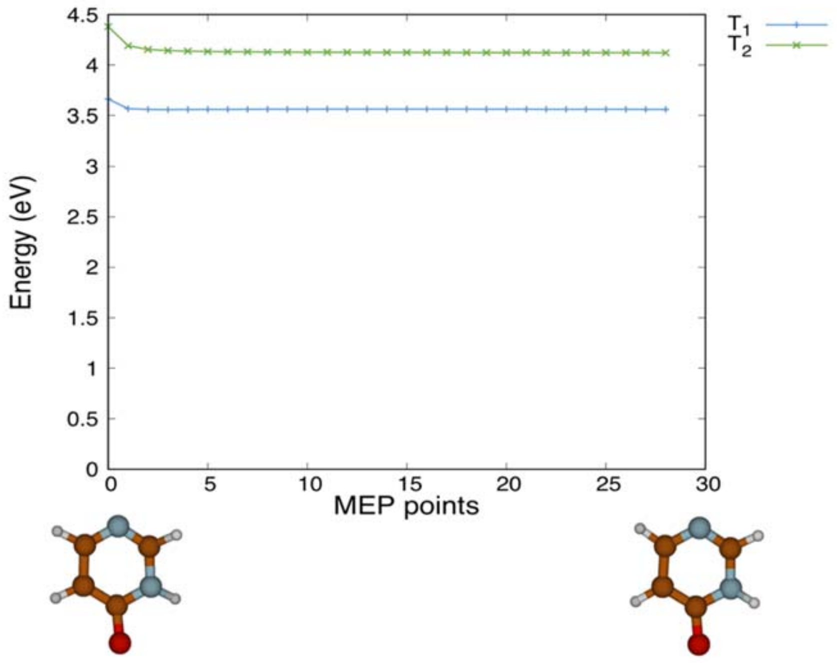

Figure S37. Minimum energy path from the $ISC_{S1/T2}$ along the T_2 excited state at XMS-CASPT2(12,9)/ANO-RCC-pVTZ level of theory.

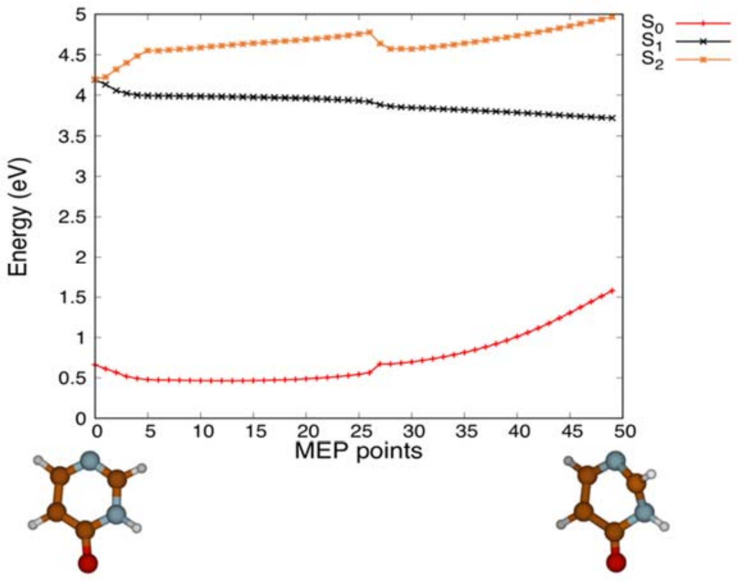

Figure S38. Minimum energy path from the $ISC_{S2/S1/T2}$ along the $S_1(\pi\pi^*)$ excited state at XMS-CASPT2(12,9)/ANO-RCC-pVTZ level of theory.

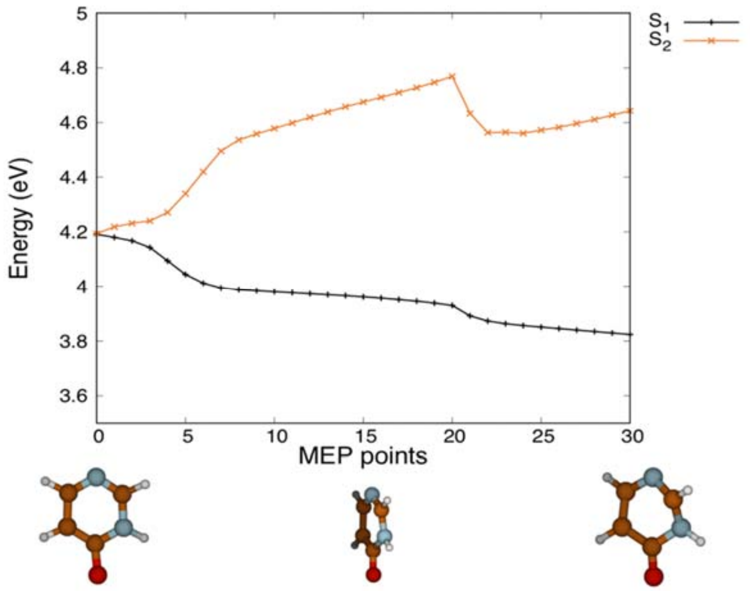

Figure S39. Minimum energy path from the $ISC_{S2/S1/T2}$ along the $S_1(n\pi^*)$ excited state at XMS-CASPT2(12,9)/ANO-RCC-pVTZ level of theory.

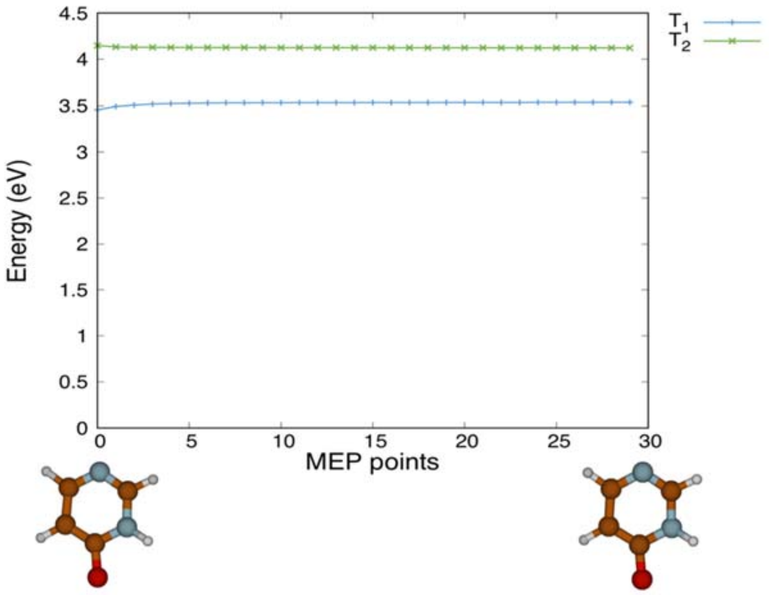

Figure S40. Minimum energy path from the $ISC_{S2/S1/T2}$ along the T_2 excited state at XMS-CASPT2(12,9)/ANO-RCC-pVTZ level of theory.

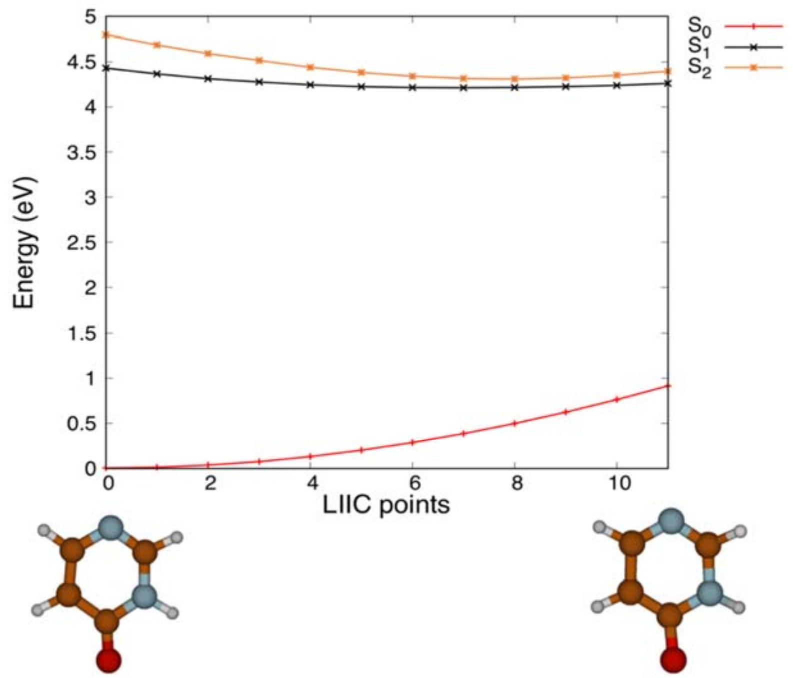

Figure S41. Linear interpolation from Franck-Condon region to the $ISC_{S1/T2}$ along $S_1(\pi\pi^*)$ excited state at XMS-CASPT2(12,9)/ANO-RCC-pVTZ level of theory.

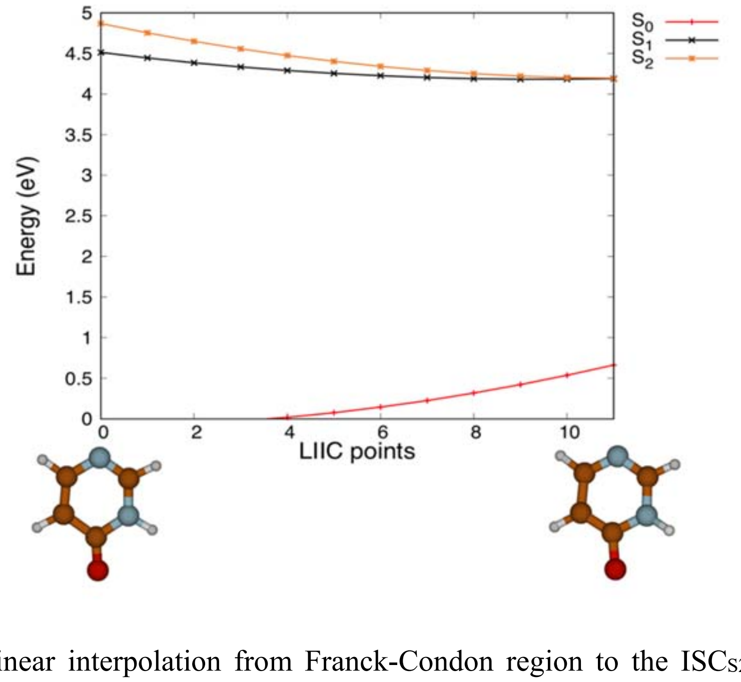

Figure S42. Linear interpolation from Franck-Condon region to the $ISC_{S2/S1/T2}$ along $S_1(\pi\pi^*)$ excited state at XMS-CASPT2(12,9)/ANO-RCC-pVTZ level of theory.

Cartesian coordinates for the stationary points

Equilibrium minimum S_0

C	1.077488	-1.120033	-0.000107
N	-0.276626	-1.104144	-0.000050
C	-1.057098	0.069316	0.000631
C	-0.224883	1.248067	0.000794
C	1.133056	1.135452	0.000708
N	1.827381	-0.056796	-0.003196
H	1.537652	-2.099333	0.000576
H	-0.714568	2.208525	-0.000549
H	1.752588	2.021152	-0.000145
O	-2.275763	-0.003630	0.001376
H	-0.796238	-1.971532	-0.000323

Minimum S_1Amin (3.69 eV)

C	1.020901	-1.061211	-0.000101
N	-0.355174	-1.153682	-0.000050
C	-0.918176	0.087007	0.434615
C	-0.079566	1.233250	0.165312
C	1.030337	1.054011	-0.731913
N	1.518201	-0.131421	-0.954499
H	1.654394	-1.844144	0.381915
H	-0.516067	2.203874	0.344519
H	1.382060	1.880501	-1.337487
O	-2.022356	0.105130	0.960036
H	-0.803696	-1.963889	0.405127

Minimum S_1Bmin (3.68 eV)

C	1.045144	-1.135344	-0.094193
N	-0.254497	-1.112056	-0.609950
C	-0.992979	-0.018797	-0.130400
C	-0.155025	1.112106	0.294437
C	1.222350	1.104431	-0.052649
N	1.789648	0.000176	-0.478916
H	1.268708	-1.588731	0.872230
H	-0.674984	2.025128	0.543980
H	1.776027	2.032679	-0.132187
O	-2.213307	-0.005665	-0.126231
H	-0.785752	-1.968696	-0.683176

Minimum T_1min (3.04 eV)

C	1.088244	-1.131214	-0.000108
N	-0.300918	-1.137080	-0.000051
C	-1.045476	0.025469	0.006012
C	-0.273451	1.246233	-0.017389
C	1.199858	1.158688	-0.018998
N	1.821851	0.012569	-0.009235
H	1.587233	-2.083583	0.009246
H	-0.808923	2.181099	-0.014795
H	1.792443	2.061890	-0.024009
O	-2.277091	-0.010019	0.031616
H	-0.815608	-2.002739	0.024537

Minimum T_2min (4.12 eV)

C	1.082754	-1.125507	-0.000107
N	-0.328096	-1.142776	-0.000050
C	-0.957739	0.094306	0.079018
C	-0.257185	1.250116	-0.061859
C	1.157365	1.147753	-0.215053
N	1.795592	-0.052713	-0.138707
H	1.544471	-2.097111	0.079337
H	-0.773306	2.196243	-0.046980
H	1.770562	2.024177	-0.341740
O	-2.271154	-0.002485	0.256997
H	-0.796137	-1.935021	0.412717

CI-$A_{S1/S0/T1}$ (3.85eV)

C	0.9220129714	-1.0074269358	0.4156972746
N	-0.2790674230	-1.1303881400	-0.3251248907
C	-1.0337725524	-0.0056373248	-0.0224773312
C	-0.1630638408	1.1130967454	0.4131360569
C	1.2217616981	1.0944474822	-0.0841656354
N	1.8048022695	-0.0385436805	-0.2386097085
H	1.3620068884	-1.8771466152	0.8761333072
H	-0.6541021915	2.0108511751	0.7584273619
H	1.6917096911	2.0064108491	-0.4381439135
O	-2.2431689666	0.0884636127	-0.1253456767
H	-0.7755129065	-2.0065272681	-0.3808938186

CI-B$_{S1/S0/T1}$ (3.70eV)

C	1.0488600100	-1.1394179195	-0.0939582395
N	-0.2657857806	-1.1078412489	-0.6131890093
C	-0.9955708014	-0.0390280378	-0.1197344801
C	-0.1453932672	1.0875336953	0.3228192271
C	1.2267177159	1.0943542499	-0.0662646883
N	1.7990667760	0.0028085755	-0.5094908682
H	1.2012784188	-1.4546055373	0.9377392790
H	-0.6626530733	1.9875063492	0.6206609219
H	1.7662059818	2.0317142488	-0.1454719374
O	-2.2148815481	0.0199012886	-0.1242314942
H	-0.7858912723	-1.9629571521	-0.7453913912

CI$_{T2/T1}$ (4.39eV)

C	1.0606838295	-1.0999412126	0.0036297626
N	-0.3757569242	-1.1460781238	-0.0082080743
C	-0.9185277633	0.1558280768	0.1454407007
C	-0.2159647104	1.2945232058	-0.0486400445
C	1.1300534021	1.1635297477	-0.2872070535
N	1.7868376203	-0.0785656194	-0.2008850167
H	1.4938783717	-2.0774649526	0.1674651534
H	-0.7025808923	2.2566536934	-0.0324566148
H	1.7651394037	2.0196919865	-0.4406619882
O	-2.2534472209	-0.0920431685	0.2718953310
H	-0.7077984172	-1.8312029945	0.6655517251

ISC$_{S2/S1/T2}$ (4.19eV)

C	1.0687245619	-1.1531769305	0.0076872151
N	-0.3311918061	-1.1193407971	0.0577421684
C	-0.9590300676	0.1134430811	0.0454990518
C	-0.2372095945	1.2570209567	-0.0762893745
C	1.2013415105	1.1231286527	-0.1488635552
N	1.8068881014	-0.0677226722	-0.1020987978
H	1.5182717335	-2.1321350765	0.0412975385
H	-0.7394852178	2.2079964722	-0.1175535843
H	1.8323527886	1.9948503661	-0.2271073809
O	-2.2765165629	0.0102559655	0.1426891318
H	-0.8665321253	-1.9306585879	0.3231337525

ISC$_{S1/T2}$ (4.39eV)

C	1.087440	-1.091290	0.007146
N	-0.324223	-1.143130	-0.024678
C	-0.998256	0.059801	-0.042587
C	-0.292668	1.226460	0.004116
C	1.141870	1.180340	0.012195
N	1.804710	0.010967	0.010906
H	1.505430	-2.047670	0.262110
H	-0.731014	2.208160	0.059265
H	1.634840	2.150110	0.055636
O	-2.320750	-0.057302	0.013291
H	-0.757587	-2.001910	-0.347500

References

(1) Arpa, E. M.; Brister, M. M.; Hoehn, S. J.; Crespo-Hernández, C. E.; Corral, I. On the Origin of the Photostability of DNA and RNA Monomers: Excited State Relaxation Mechanism of the Pyrimidine Chromophore. *J. Phys. Chem. Lett.* **2020**, *11*, 5156–5161.

(2) Reichardt, C.; Vogt, R. A.; Crespo-Hernández, C. E. On the Origin of Ultrafast Nonradiative Transitions in Nitro-Polycyclic Aromatic Hydrocarbons: Excited-State Dynamics in 1-Nitronaphthalene. *J. Chem. Phys.* **2009**, *131*, 224518.

(3) Krul, S. E.; Hoehn, S. J.; Feierabend, K. J.; Crespo-Hernández, C. E. Excited State Dynamics of 7-Deazaguanosine and Guanosine 5′-Monophosphate. *J. Chem. Phys.* **2021**, *154*, 075103.

(4) Snellenburg, J. J.; Laptenok, S.; Seger, R.; Mullen, K. M.; van Stokkum, I. H. M. Glotaran: A Java-Based Graphical User Interface for the R Package TIMP. *J. Stat. Softw.* **2012**, *49*, 1–22.

(5) Scuseria, G. E.; Janssen, C. L.; Schaefer, H. F. An Efficient Reformulation of the Closed-shell Coupled Cluster Single and Double Excitation (CCSD) Equations. *J. Chem. Phys.* **1998**, *89*, 7382.

(6) Woon, D. E.; Dunning, T. H. Gaussian Basis Sets for Use in Correlated Molecular Calculations. IV. Calculation of Static Electrical Response Properties. *J. Chem. Phys.* **1994**, *100*, 2975.

(7) Kendall, R. A.; Dunning, T. H.; Harrison, R. J. Electron Affinities of the First-row Atoms Revisited. Systematic Basis Sets and Wave Functions. *J. Chem. Phys.* **1992**, *96*, 6796–6806.

(8) Marenich, A. V.; Cramer, C. J.; Truhlar, D. G. Universal Solvation Model Based on Solute Electron Density and on a Continuum Model of the Solvent Defined by the Bulk Dielectric Constant and Atomic Surface Tensions. *J. Phys. Chem. B* **2009**, *113*, 6378–6396.

(9) Wilson, A. K.; van Mourik, T.; Dunning, T. H. Gaussian Basis Sets for Use in Correlated Molecular Calculations. VI. Sextuple Zeta Correlation Consistent Basis Sets for Boron through Neon. *J. Mol. Struct. THEOCHEM* **1996**, *388*, 339–349.

(10) Neese, F. The ORCA Program System. *WIRES Comput. Mol. Sci.* **2012**, *2*, 73–78.

(11) Shiozaki, T.; Gyroffy, W.; Celani, P.; Werner, H. J. Communication: Extended Multi-State Complete Active Space Second-Order Perturbation Theory: Energy and Nuclear Gradients. *J. Chem. Phys.* **2011**, *135*, 081106.

(12) Park, J. W.; Shiozaki, T. Analytical Derivative Coupling for Multistate CASPT2 Theory. *J. Chem. Theory Comput.* **2017**, *13*, 2561–2570.

(13) Shiozaki, T. BAGEL: Brilliantly Advanced General Electronic-structure Library. *WIREs Comput. Mol. Sci.* **2018**, *8*, 1331.

(14) Reiher, M.; Wolf, A. Exact Decoupling of the Dirac Hamiltonian. I. General Theory. *J. Chem. Phys.* **2004**, *121*, 2037.

(15) Peng, D.; Reiher, M. Exact Decoupling of the Relativistic Fock Operator. *Theor. Chem. Acc.* **2012**, *131*, 1–20.

(16) Reiher, M.; Wolf, A. Exact Decoupling of the Dirac Hamiltonian. II. The Generalized Douglas–Kroll–Hess Transformation up to Arbitrary Order. *J. Chem. Phys.* **2004**, *121*, 10945.

(17) Pierloot, K.; Dumez, B.; Widmark, P.-O.; Roos, B. O. Density Matrix Averaged

Atomic Natural Orbital (ANO) Basis Sets for Correlated Molecular Wave Functions. *Theor. Chim. Acta.* **1995**, *90*, 87–114.
(18) Woon, D. E.; Dunning, T. H. Gaussian Basis Sets for Use in Correlated Molecular Calculations. V. Core-valence Basis Sets for Boron through Neon. *J. Chem. Phys.* **1995**, *103*, 4572–4585.
(19) Hjorth Larsen, A.; Jørgen Mortensen, J.; Blomqvist, J.; Castelli, I. E.; Christensen, R.; Dułak, M.; Friis, J.; Groves, M. N.; Hammer, B.; Hargus, C.; Hermes, E. D.; Jennings, P. C.; Bjerre Jensen, P.; Kermode, J.; Kitchin, J. R.; Leonhard Kolsbjerg, E.; Kubal, J.; Kaasbjerg, K.; Lysgaard, S.; Bergmann Maronsson, J.; Maxson, T.; Olsen, T.; Pastewka, L.; Peterson, A.; Rostgaard, C.; Schiøtz, J.; Schütt, O.; Strange, M.; Thygesen, K. S.; Vegge, T.; Vilhelmsen, L.; Walter, M.; Zeng, Z.; Jacobsen, K. W. The Atomic Simulation Environment—a Python Library for Working with Atoms. *J. Phys. Condens. Matter.* **2017**, *29*, 273002.
(20) Keal, T. W.; Koslowski, A.; Thiel, W. Comparison of Algorithms for Conical Intersection Optimisation Using Semiempirical Methods. *Theor. Chem. Accounts.* **2007**, *118*, 837–844.
(21) Levine, B. G.; Coe, J. D.; Martínez, T. J. Optimizing Conical Intersections without Derivative Coupling Vectors: Application to Multistate Multireference Second-Order Perturbation Theory (MS-CASPT2). *J. Phys. Chem. B.* **2008**, *112*, 405–413.
(22) Fdez. Galván, I.; Vacher, M.; Alavi, A.; Angeli, C.; Aquilante, F.; Autschbach, J.; Bao, J. J.; Bokarev, S. I.; Bogdanov, N. A.; Carlson, R. K.; Chibotaru, L. F.; Creutzberg, J.; Dattani, N.; Delcey, M. G.; Dong, S. S.; Dreuw, A.; Freitag, L.; Frutos, L. M.; Gagliardi, L.; Gendron, F.; Giussani, A.; González, L.; Grell, G.; Guo, M.; Hoyer, C. E.; Johansson, M.; Keller, S.; Knecht, S.; Kovačević, G.; Källman, E.; Li Manni, G.; Lundberg, M.; Ma, Y.; Mai, S.; Malhado, J. P.; Malmqvist, P. Å.; Marquetand, P.; Mewes, S. A.; Norell, J.; Olivucci, M.; Oppel, M.; Phung, Q. M.; Pierloot, K.; Plasser, F.; Reiher, M.; Sand, A. M.; Schapiro, I.; Sharma, P.; Stein, C. J.; Sørensen, L. K.; Truhlar, D. G.; Ugandi, M.; Ungur, L.; Valentini, A.; Vancoillie, S.; Veryazov, V.; Weser, O.; Wesołowski, T. A.; Widmark, P. O.; Wouters, S.; Zech, A.; Zobel, J. P.; Lindh, R. OpenMolcas: From Source Code to Insight. *J. Chem. Theory Comput.* **2019,** *12*, 5925–5964.
(23) Verlet, L. Computer "Experiments" on Classical Fluids. I. Thermodynamical Properties of Lennard-Jones Molecules. *Phys. Rev.* **1967**, *159*, 98.
(24) Verlet, L. Computer "Experiments" on Classical Fluids. II. Equilibrium Correlation Functions. *Phys. Rev.* **1968**, *165*, 201–214.
(25) Granucci, G.; Persico, M.; Zoccante, A. Including Quantum Decoherence in Surface Hopping. *J. Chem. Phys.* **2010**, *133*, 134111.
(26) Richter, M.; Marquetand, P.; González-Vázquez, J.; Sola, I.; González, L. SHARC: Ab Initio Molecular Dynamics with Surface Hopping in the Adiabatic Representation Including Arbitrary Couplings. *J. Chem. Theory Comput.* **2011**, *7*, 1253–1258.
(27) Petersen, J.; Mitrić, R. Electronic Coherence within the Semiclassical Field-Induced Surface Hopping Method: Strong Field Quantum Control in K2. *Phys. Chem. Chem. Phys.* **2012**, *14*, 8299–8306.
(28) Izsák, R.; Neese, F. An Overlap Fitted Chain of Spheres Exchange Method. *J. Chem. Phys.* **2011**, *135*, 144105.
(29) Neese, F.; Wennmohs, F.; Hansen, A.; Becker, U. Efficient, Approximate and Parallel Hartree–Fock and Hybrid DFT Calculations. A 'Chain-of-Spheres'

Algorithm for the Hartree–Fock Exchange. *Chem. Phys.* **2009**, *356*, 98–109.
(30) Becke, A. D. Density-Functional Thermochemistry. III. The Role of Exact Exchange. *J. Chem. Phys.* **1993**, *98*, 5648.
(31) Martyna, G. J.; Klein, M. L.; Tuckerman, M. Nosé–Hoover Chains: The Canonical Ensemble via Continuous Dynamics. *J. Chem. Phys.* **1998**, *97*, 2635.
(32) Franzen, S.; Skalski, B.; Bartolotti, L.; Delley, B. The Coupling of Tautomerization to Hydration in the Transition State on the Pyrimidine Photohydration Reaction Path. *Phys. Chem. Chem. Phys.* **2014**, *16*, 20164–20174.
(33) Allouche, A. R. Gabedit—A Graphical User Interface for Computational Chemistry Softwares. *J. Comput. Chem.* **2011**, *32*, 174–182.
